# Supplementary material for: Increased uptake of tuberculosis preventive therapy (TPT) among people living with HIV following the 100-days accelerated campaign: A retrospective review of routinely collected data at six urban public health facilities in Uganda
Source: PLoS One. 2023 Feb 23;18(2):e0268935. doi: 10.1371/journal.pone.0268935 (PMC9949662; doi:10.1371/journal.pone.0268935)
Supplement: S2 File — (DOCX) [file pone.0268935.s002.docx]

# **Supplementary 2: Additional information and analysis**

**S2 Appendix 1**

**Data variables**

The following variables were extracted from the Uganda EMR database: Patients’ socio-demographics (age, birthdates, gender, marital status), clinical factors (clinic visit date, weight in kilograms, height in meters, ART status at IPT initiation, ART start date, WHO stage, HIV/RNA viral load (absolute), pregnancy status, TB treatment history, Isoniazid prophylaxis start date, and place of residence.

The following information were extracted from the TPT registers: IPT initiation date, IPT side effects, IPT outcome (completed 6-month IPT dose, loss to follow-up, died, stopped, transferred to another health facility), medical reasons for stopping IPT (including side effects, developed active TB).

S2 Table X1: 100 days IPT campaign outline of activities and timelines

| Action | Time line |
| --- | --- |
| Update the DHT about the 100 day TPT scale up strategy | 2nd July 2019 |
| Collaborate with the DHT to plan and execute the TPT rapid response | 1st July 2019 |
| Analyse site specific gaps and work with district leaderships in addressing them through CQI projects that are to be reviewed on a weekly basis | Weekly till Sept 30th |
| Facilitate weekly collection and submission of TPT enrollments and completion data into the surge dash board | Weekly till Sept 30th |
| Support active safety monitoring for INH and reporting through the NDA system | Daily |
| Collection and monthly submission of INH stock status data to the national level and fast tracking deliveries and redistribution of excess stock | Monthly |
| Weekly feedback to facility teams on initiation and completion rates ( data utilization) | Weekly |
| Use of stickers to mark the files of clients that are on INH so as to avoid missed opportunities | Daily |

S2 Table X2: Checklist for screening contraindications before initiation of TPT

| **s/n** | **Contraindication** | **Yes (tick)** | **No**  **(tick)** |
| --- | --- | --- | --- |
| 1 | Has the patient got known or suspected hypersensitivity reaction to Isoniazid? Symptoms may include: difficulty in breathing, swelling of the lips, tongue or face |  |  |
| 2 | Has the patient got any symptoms of active hepatitis (jaundice, nausea, vomiting, right upper abdominal pain, dark urine, pale stools)? |  |  |
| 3 | Is the patient an alcoholic (regular or heavy alcohol consumption)? |  |  |
| 4 | Does the patient have history of convulsions? (excluding febrile convulsions) |  |  |
| 5 | Does the patient have history of mental illness? |  |  |
| 6 | Does the patient have peripheral neuropathy (burning sensation in the limbs)? |  |  |
| 7 | Is the patient taking any medications: oral Ketoconazole, Itraconazole, Phenytoin, Carbamazepine, Warfarin, Theophylline, Disulfiram, selective serotonin re-uptake inhibitor antidepressants (e.g. Citalopram, Fluoxetine, Paroxetine, Sertraline) |  |  |

**S2 Appendix 2**

**S2 Table 1: Detailed IPT outcomes by participants’ baseline characteristics at TPT start**

| Characteristics | Treatment outcomes | | | | | |  |  |
| --- | --- | --- | --- | --- | --- | --- | --- | --- |
|  | Completed  n (%) | Died  n (%) | LTFU  n (%) | Stopped  n (%) | Outcome not indicated  n (%) | Transferred out  n (%) | Total  n (%) | P value |
| Year and quarter of TPT initiation |  |  |  |  |  |  |  |  |
| 2016 Q1 | 60 (90.9) | 0 | 4 (6.1) | 1 (1.5) | 0 | 1 (1.5) | 66 (100.0) | <0.001 |
| 2016 Q2 | 90 (81.1) | 1 (0.9) | 12 (10.8) | 0 | 0 | 8 (7.2) | 111 (100.0) |  |
| 2016 Q3 | 69 (84.2) | 0 | 12 (14.6) | 0 | 1 (1.2) | 0 | 82 (100.0) |  |
| 2016 Q4 | 314 (68.6) | 3 (0.7) | 98 (21.4) | 10 (2.2) | 14 (3.1) | 19 (4.2) | 458 (100.0) |  |
| 2017 Q1 | 76 (59.4) | 0 | 44 (34.4) | 3 (2.3) | 0 | 5 (3.9) | 128 (100.0) |  |
| 2017 Q2 | 179 (66.3) | 2 (0.7) | 57 (21.1) | 9 (3.3) | 14 (5.2) | 9 (3.3) | 270 (100.0) |  |
| 2017 Q3 | 313 (70.8) | 0 | 75 (17.0) | 7 (1.6) | 35 (7.9) | 12 (2.7) | 442 (100.0) |  |
| 2017 Q4 | 271 (74.9) | 1 (0.3) | 50 (13.8) | 19 (5.3) | 6 (1.7) | 15 (4.1) | 362 (100.0) |  |
| 2018 Q1 | 15 (83.3) | 0 | 0 | 1 (5.6) | 0 | 2 (11.1) | 18 (100.0) |  |
| 2018 Q2 | 74 (77.9) | 0 | 10 (10.5) | 4 (4.2) | 2 (2.1) | 5 (5.3) | 95 (100.0) |  |
| 2018 Q3 | 46 (61.3) | 0 | 17 (22.7) | 1 (1.3) | 3 (4.0) | 8 (10.7) | 75 (100.0) |  |
| 2018 Q4¶ | 248 (79.7) | 2 (0.6) | 43 (13.8) | 5 (1.6) | 2 (0.6) | 11 (3.5) | 311(100.0) |  |
| 2019 Q1 | 3314 (93.6) | 36 (1.0) | 106 (3.0) | 20 (0.6) | 22 (0.6) | 43 (1.2) | 3541 (100.0) |  |
| 2019 Q2 | 3777 (96.0) | 3 (0.1) | 90 (2.3) | 19 (0.5) | 9 (0.2) | 38 (1.0) | 3936 (100.0) |  |
| 2019 Q3 | 233 (98.7) | 0 | 2 (0.9) | 0 | 1 (0.4) | 0 | 236 (100.0) |  |
| Sex |  |  |  |  |  |  |  |  |
| Male | 2557 (91.5) | 15 (0.6) | 143 (5.1) | 23 (0.8) | 18 (0.6) | 38 (1.4) | 2794 (100.0) | 0.002 |
| Female | 6522 (88.9) | 33 (0.5) | 477 (6.5) | 76 (1.0) | 91 (1.2) | 138 (1.9) | 7337 (100.0) |  |
| Age in years at IPT start |  |  |  |  |  |  |  |  |
| 15 – 24 | 982 (76.4) | 5 (0.4) | 181 (14.1) | 17 (1.3) | 41 (3.2) | 59 (4.6) | 1285 (100.0) | <0.001 |
| 25 – 34 | 3652 (87.8) | 25 (0.6) | 305 (7.3) | 43 (1.0) | 51 (1.2) | 85 (2.1) | 4161 (100.0) |  |
| ≥ 35 | 4445 (94.9) | 18 (0.4) | 134 (2.9) | 39 (0.8) | 17 (0.3) | 32 (0.7) | 4685 (100.0) |  |
| ART status at IPT |  |  |  |  |  |  |  |  |
| Not on ART | 244 (65.4) | 3 (0.8) | 90 (24.1) | 6 (1.6) | 6 (1.6) | 24 (6.5) | 373 (100.0) | <0.001 |
| Newly on ART^†^ | 2290 (77.5) | 16 (0.6) | 406 (13.7) | 57 (1.9) | 72 (2.4) | 116 (3.9) | 2957 (100.0) |  |
| ART experienced^††^ | 6545 (96.3) | 29 (0.4) | 124 (1.8) | 36 (0.5) | 31 (0.5) | 36 (0.5) | 6801 (100.0) |  |
| WHO HIV stage |  |  |  |  |  |  |  |  |
| Stage 1 or 2 | 8767 (89.9) | 45 (0.5) | 581 (6.0) | 92 (0.9) | 104 (1.1) | 162 (1.7) | 9751 (100.0) | 0.014 |
| Stage 3 or 4 | 239 (84.8) | 3 (1.1) | 23 (8.2) | 7 (2.5) | 2 (0.7) | 8 (2.8) | 282 (100.0) |  |
| BMI at IPT start (kgs/m^2^) |  |  |  |  |  |  |  |  |
| <18.5 | 801 (89.0) | 7 (0.8) | 58 (6.4) | 15 (1.7) | 7 (0.8) | 12 (1.3) | 900 (100.0) | 0.086 |
| ≥18.5 | 7961 (90.1) | 37 (0.4) | 514 (5.8) | 78 (0.9) | 100 (1.1) | 145 (1.6) | 8835 (100.0) |  |
| Pregnant during IPT |  |  |  |  |  |  |  |  |
| No | 2525 (91.1) | 26 (0.4) | 310 (5.4) | 57 (1.0) | 39 (0.7) | 80 (1.4) | 5764 (100.0) | <0.001 |
| Yes | 1270 (80.7) | 7 (0.5) | 167 (10.6) | 19 (1.2) | 52 (3.3) | 58 (3.7) | 1573 (100.0) |  |

† Newly on ART defined as being on ART for less than 3 months at IPT start

†† ART experienced defined as being on ART for more than 3 months at IPT start

**S2 Table 2 Proportion of TPT uptake across participants characteristics by year (un adjusted)**

| **Factors** | **2016**  **N=33,021** | **2017**  **N=35,718** | **2018**  **N=36,198** | **2019**  **N=37,418** |
| --- | --- | --- | --- | --- |
|  | % (n/N) | % (n/N) | % (n/N) | % (n/N) |
| **Demographic factors** |  |  |  |  |
| **Sex** |  |  |  |  |
| Male | 5.7 (475/8,358) | 4.4 (396/8,923) | 2.9 (268/9,315) | 67.1 (6,577/9,800) |
| Female | 4.5 (1,108/24,663) | 4.6 (1,236/26,795) | 2.4 (644/26,883) | 68.4 (18,913/27,618) |
| *P-value* | *<0.001* | *0.493* | *0.011* | *0.013* |
|  |  |  |  |  |
| **Age in years** |  |  |  |  |
| 15 – 19 | 7.0 (39/558) | 11.8 (67/570) | 11.4 (63/551) | 79.9 (426/533) |
| 20 – 24 | 6.7 (193/2,863) | 10.5 (320/3036) | 6.2 (166/2,694) | 73.1 (1,956/2,676) |
| 25 – 34 | 5.3 (706/13,357) | 5.4 (767/14,342) | 3.0 (424/14,139) | 70.5 (10,001/14,181) |
| 35+ | 4.0 (645/16,243) | 2.7 (478/17,770) | 1.4 (259/18,814) | 65.4 (13,107/20,028) |
| *P-value* | *<0.001* | *<0.001* | *<0.001* | *<0.001* |
|  |  |  |  |  |
| **Clinical factors** |  |  |  |  |
| **On ART** |  |  |  |  |
| No | 27.9 (616/2,209) | 13.4 (206/1,533) | 0.9 (7/781) | 52.5 (322/613) |
| Yes | 3.1 (967/30,812) | 4.2 (1,426/34,185) | 2.6 (905/35,417) | 68.4 (25,168/36,805) |
| *P-value* | *<0.001* | *<0.001* | *0.003* | *<0.001* |
|  |  |  |  |  |
| WHO stage |  |  |  |  |
| 1 or 2 | 4.8 (1,417/29,530) | 4.7 (1,553/33,074) | 2.5 (877/34,855) | 68.2 (24,867/36,442) |
| 3 or 4 | 4.8 (166/3,491) | 3.0 (79/2,644) | 2.6 (35/1,343) | 63.8 (623/976) |
| *P-value* | *0.910* | *<0.001* | *0.836* | *0.004* |
|  |  |  |  |  |
| BMI kg/m2 |  |  |  |  |
| <18.5 | 5.5 (211/3,811) | 4.2 (162/3,854) | 3.2 (118/3,650) | 65.6 (2,259/3,444) |
| ≥18.5 | 4.7 (1,372/29,210) | 4.6 (1,470/31,864) | 2.4 (794/32,548) | 68.4 (23,231/33,974) |
| *P-value* | *0.023* | *0.250* | *0.004* | *0.001* |
|  |  |  |  |  |
| TB treatment history |  |  |  |  |
| No | 4.8 (1,583/33,021) | 4.6 (1,629/35,314) | 2.6 (909/35,301) | 68.3 (24,714/36,187) |
| Yes | 0 | 0.7 (3/404) | 0.3 (3/897) | 63.0 (776/1,231) |
| *P-value* |  | *<0.001* | *<0.001* | *<0.001* |

N denotes number of patients visited clinic during a specific year, n denotes number initiated on TPT. Numbers analyzed only included complete cases on all factors same as what was analyzed in the model for TPT uptake presented in Table 2.

**S2 Table 3a TPT uptake by clinic over the 4 years**

|  | 2016 | | | 2017 | | | 2018 | | | 2019 | | |
| --- | --- | --- | --- | --- | --- | --- | --- | --- | --- | --- | --- | --- |
| Health facility¶ | Total patients | TPT uptake | | Total patients | TPT uptake | | Total patients | TPT uptake | | Total patients | TPT uptake | |
|  |  | number | % |  | number | % |  | number | % |  | number | % |
| A | 6,062 | 119 | 1.96 | 5,972 | 333 | 5.58 | 5,753 | 122 | 2.12 | 5,705 | 3,555 | 62.31 |
| B | 8,827 | 155 | 1.76 | 9,091 | 397 | 4.37 | 9,019 | 260 | 2.88 | 8,999 | 6,711 | 74.57 |
| C | 4,113 | 68 | 1.65 | 4,408 | 180 | 4.08 | 4,698 | 210 | 4.47 | 4,699 | 2,833 | 60.29 |
| D | 6,468 | 283 | 4.38 | 6,685 | 168 | 2.51 | 7,023 | 114 | 1.62 | 7,201 | 4,318 | 59.96 |
| E | 2,533 | 103 | 4.07 | 2,674 | 214 | 8.00 | 2,623 | 155 | 5.91 | 2,408 | 1,739 | 72.22 |
| F | 10,679 | 996 | 9.33 | 10,770 | 421 | 3.91 | 11,211 | 94 | 0.84 | 11,475 | 6,942 | 60.50 |
| Total | 38,682 | 1,724 | 4.46 | 39,600 | 1,713 | 4.33 | 40,327 | 955 | 2.37 | 40,487 | 26,098 | 64.46 |

¶ Health facilities are anonymously labelled to ensure confidentiality

**S2 Table 3b TPT completion by clinic over the 4 years**

|  | 2016 | | | 2017 | | | 2018 | | | 2019 | | |
| --- | --- | --- | --- | --- | --- | --- | --- | --- | --- | --- | --- | --- |
| Health facility | Total patients§ | TPT completion | | Total patients§ | TPT completion | | Total patients§ | TPT completion | | Total patients§ | TPT completion | |
|  |  | number | % |  | number | % |  | number | % |  | number | % |
| A | 82 | 68 | 82.9 | 188 | 110 | 58.5 | 54 | 49 | 90.7 | 887 | 870 | 98.1 |
| B | 109 | 67 | 61.5 | 301 | 229 | 76.1 | 148 | 129 | 87.2 | 1,910 | 1,849 | 96.8 |
| C | 30 | 28 | 93.3 | 136 | 106 | 77.9 | 159 | 120 | 75.5 | 852 | 810 | 95.1 |
| D | 207 | 184 | 88.9 | 115 | 100 | 87.0 | 73 | 63 | 86.3 | 1,591 | 1,519 | 95.5 |
| E | 75 | 37 | 49.3 | 161 | 130 | 80.7 | 31 | 22 | 71.0 | 549 | 533 | 97.1 |
| F | 171 | 149 | 87.1 | 205 | 164 | 80.0 | 1 | 0 | 0.0 | 1,811 | 1,743 | 96.2 |
| Total | 674 | 533 | 79.1 | 1,106 | 839 | 75.9 | 466 | 383 | 82.2 | 7,600 | 7,324 | 96.4 |

¶ Health facilities are anonymously labelled in order to preserve confidentiality

§ Total patients in this table excludes those who had missing outcome (i.e. TPT completion) data. Missing values on TB completion: overall (n=285/10131), and by health facility: A (n=11), B (n=99), C (n=42), D (n=44), E (n=26), and F (n=63).

S2 Table 4: TPT completion and associated factors – sensitivity analyses: Worst-case scenarios, Best-case scenarios and multiple imputation using chained equations (MICE)

| **Factor** | **Worst-case scenario**  **N=** **9,797^†^** | | **Best-case scenario**  **N=** **9,797^††^** | | **Multiple imputation**  **N=** **9,895^†††^** | |
| --- | --- | --- | --- | --- | --- | --- |
| Calendar year and quarter | Adjusted  **PR (95%CI)** | **P-value** | Adjusted  **PR (95%CI)** | **P-value** | Adjusted  **PR (95%CI)** | **P-value** |
| 2016 Q1 | 1.15 (1.11,1.19) | <0.001 | 1.11 (1.05,1.17) | <0.001 | 1.12 (1.06,1.18) | <0.001 |
| 2016 Q2 | 1.04 (0.90,1.20) | 0.636 | 1.07 (0.90,1.26) | 0.452 | 1.05 (0.88,1.24) | 0.593 |
| 2016 Q3 | 1.06 (1.02,1.10) | 0.004 | 1.02 (0.97,1.06) | 0.407 | 1.03 (0.98,1.08) | 0.188 |
| 2016 Q4 | 0.91 (0.75,1.10) | 0.322 | 0.93 (0.78,1.11) | 0.405 | 0.92(0.76,1.11) | 0.406 |
| 2017 Q1 | 0.80 (0.63,1.00) | 0.053 | 0.79 (0.61,1.01) | 0.065 | 0.77 (0.60,0.99) | 0.048 |
| 2017 Q2 | 0.84 (0.76,0.93) | 0.001 | 0.90 (0.80,1.00) | 0.058 | 0.89 (0.80,0.99) | 0.032 |
| 2017 Q3 | 0.89 (0.82,0.97) | 0.006 | 0.98 (0.89,1.07) | 0.603 | 0.96 (0.87,1.06) | 0.400 |
| 2017 Q4 | 0.95 (0.86,1.05) | 0.330 | 0.97 (0.90,1.04) | 0.399 | 0.96 (0.89,1.05) | 0.375 |
| 2018 Q1 | 1.09 (0.96,1.24) | 0.180 | 1.15 (1.10,1.22) | <0.001 | 1.15 (1.06,1.25) | 0.001 |
| 2018 Q2 | 0.96 (0.90,1.03) | 0.292 | 1.01 (0.96,1.06) | 0.801 | 1.00 (0.93,1.07) | 0.981 |
| 2018 Q3 | 0.79 (0.67,0.92) | 0.003 | 0.92 (0.73,1.17) | 0.517 | 0.91 (0.75,1.09) | 0.304 |
| 2018 Q4¶ | 1 |  | 1 |  | 1 |  |
| 2019 Q1 | 1.09 (1.04,1.14) | 0.001 | 1.08 (1.03,1.13) | 0.001 | 1.08 (1.03,1.14) | 0.002 |
| 2019 Q2 | 1.10 (1.04,1.16) | <0.001 | 1.09 (1.04,1.14) | <0.001 | 1.09 (1.04,1.15) | 0.001 |
| **Demographic factors** |  |  |  |  |  |  |
| Sex |  |  |  |  |  |  |
| Male | 1 |  | 1 |  | 1 |  |
| Female | 0.99 (0.97, 1.00) | 0.129 | 0.99 (0.98, 1.00) | 0.188 | 0.99 (0.98, 1.00) | 0.149 |
| Age in years, n (%) |  |  |  |  |  |  |
| 15 – 19 | 1 |  | 1 |  | 1 |  |
| 20 – 24 | 1.06 (0.98, 1.16) | 0.161 | 1.06 (0.98, 1.15) | 0.171 | 1.06 (0.98, 1.15) | 0.119 |
| 25 – 34 | 1.14 (1.05, 1.25) | 0.003 | 1.09 (0.99, 1.21) | 0.078 | 1.11 (1.02, 1.22) | 0.021 |
| 35+ | 1.18 (1.08, 1.30) | <0.001 | 1.12 (1.01, 1.24) | 0.038 | 1.14 (1.03, 1.25) | 0.008 |
| **Clinical factors** |  |  |  |  |  |  |
| ART status |  |  |  |  |  |  |
| Not on ART | 0.80 (0.69, 0.92) | 0.002 | 0.86 (0.75, 0.98) | 0.023 | 0.82 (0.72, 0.94) | 0.004 |
| Newly on ART^‡^ | 0.91 (0.86, 0.96) | 0.002 | 0.94 (0.91, 0.97) | 0.001 | 0.93 (0.90, 0.97) | <0.001 |
| ART experienced | 1 |  | 1 |  | 1 |  |
| WHO stage |  |  |  |  |  |  |
| 1 or 2 | 1 |  | 1 |  | 1 |  |
| 3 or 4 | 0.97 (0.92, 1.02) | 0.203 | 0.98 (0.94, 1.01) | 0.169 | 0.97 (0.93, 1.00) | 0.089 |

† At worst-case scenario sensitivity analysis, we assumed that participants who had missing outcome considered as never completed TPT. Other covariates that had missing values were modelled with their missing values.

†† At best-case scenario sensitivity analysis, we assumed that participants who had missing outcome considered as completed TPT. Other covariates with missing values were modelled with their missing values

††† Multiple imputation using chained equations (MICE) was performed for completing missing values on: outcome [TPT completion (n=284/9,895, 3%) and WHO stage (n=98/9,895, 1%).

**PR** – Prevalence Ratio estimated using modified Poisson regression model with cluster standard errors to account for clustering since data was collected from different clinics.

**CI** – confidence interval, **Q** – calendar quarter

‡ Newly on ART includes participants who were on ART for ≤ 3 months at TPT initiation.
