# Supplementary material for: Increased uptake of tuberculosis preventive therapy (TPT) among people living with HIV following the 100-days accelerated campaign: A retrospective review of routinely collected data at six urban public health facilities in Uganda
Source: PLoS One. 2023 Feb 23;18(2):e0268935. doi: 10.1371/journal.pone.0268935 (PMC9949662; doi:10.1371/journal.pone.0268935)
Supplement: S3 File — (DOCX) [file pone.0268935.s003.docx]

**USING ROUTINELY COLLECTED SECONDARY DATA TO ESTIMATE THE UPTAKE AND EFFECT OF ISONIAZID PREVENTIVE THERAPY ON TUBERCULOSIS INCIDENCE AND PREGNANCY OUTCOMES AMONG PEOPLE LIVING WITH HIV RECEIVING CARE IN PUBLIC HEALTH FACILITIES IN UGANDA**

**PATIENT’S LEVEL DATA COLLECTION FORM V1.0.2**

**Site code | Participant number**

***Write; Site Code (1 digit) + Patient’s number (3 digits).***

***Eg: “1 001”. Meaning “Kitebi – patient number 001”***

**Patient study number**

| **Form 0: General Information** | |
| --- | --- |
| **Data collection details** |  |
| Facility name | 1. Kiswa HC III  2. Kawaala HC III  3. Komamboga HC III  4. Kitebi HC III  5. Kisugu HC III  6. Kisenyi HC IV |
| Date of collection (dd/mm/yyyy): | ___________/__________/2020 |
| **Research Assistant details** |  |
| Name |  |
| Code |  |
| Signature |  |

**FORM 1: ISONIAZID PREVENTIVE THERAPY (IPT) INFORMATION (*Data source: NTLP IPT register*)**

**Note: IPT information for HIV+ patients initiated on IPT dose during 2016 and 2019**

| **General Patient’s Information** | |
| --- | --- |
| **Patient’s HIV clinic number (IDCNO)** |  |
| **Cohort Year** |  |
| **Cohort Month** |  |
| **IPT serial number/year** |  |
| **Age (in years)** |  |
| **Sex** | 1. Male 2. Female |
| **Address** | District: |
|  | County: |
|  | Sub-county: |
|  | Parish: |
|  | LC1 village: |
|  | Nearest health unit: |
| **HIV status** | 1. HIV pos (CT1) 2. HIV neg (CT2) 3. HIV status unknown (Un) |
| **Entry point** | 1. HIV clinic 2. ANC clinic 3. TB clinic 4. Other (specify) |
| **IPT Initiation** | |
| **Date (dd/mm/yyyy)** | __________/__________/____________ |
| **Weight (Kg)** |  |
| **Isoniazid dose (mg)** |  |
| **IPT Follow-Ups** | |
| **End of 1^st^ 2weeks of therapy** | |
| **Visit date (day/month)** | ______/____________ |
| **Appointment kept** | 1. YES 2. NO |
| **INH Received** | 1. YES 2. NO |
| **Adherence score** | 1. Good (>95%) 2. Fair (85-95%) 3. Poor (<85%) |
| **Reasons for poor adherence** | 1. Side effects 2. Illness/hospital 3. Drugs stock-outs  4. Patient lack finances 5. Other patient’s reason |
| **TB status** | 1. No signs 2. Presumptive TB case  3. Diagnosed with TB 4. On TB treatment |
| **Weight (Kgs)** | _______________________________________________ |
| **Side effects** | 1. YES 2. NO |
| **Specify side effects** | 1. Numbness, tingling & burning sensation  2. Blurred or loss of vision 3. Convulsion  4. Mood change 5. Unusual bleeding 6. Skin rash  7. Sore throat 8. Joint pain 9. Fever 10. Dizzyness  11. Loss of appetite, nausea & vomiting 12. Dark urine  13. Other (specify)________________ |
| **End of 1^st^ month of therapy** | |
| **Visit date (day/month)** | ______/____________ |
| **Appointment kept** | 1. YES 2. NO |
| **INH Received** | 1. YES 2. NO |
| **Adherence score** | 1. Good (>95%) 2. Fair (85-95%) 3. Poor (<85%) |
| **Reasons for poor adherence** | 1. Side effects 2. Illness/hospital 3. Drugs stock-outs  4. Patient lack finances 5. Other patient’s reason |
| **TB status** | 1. No signs 2. Presumptive TB case  3. Diagnosed with TB 4. On TB treatment |
| **Weight (Kgs)** | _______________________________________________ |
| **Side effects** | 1. YES 2. NO |
| **Specify side effects** | 1. Numbness, tingling & burning sensation  2. Blurred or loss of vision 3. Convulsion  4. Mood change 5. Unusual bleeding 6. Skin rash  7. Sore throat 8. Joint pain 9. Fever 10. Dizziness  11. Loss of appetite, nausea & vomiting 12. Dark urine  13. Other (specify)________________ |
| **End of 2^nd^ month of therapy** | |
| **Visit date (day/month)** | ______/____________ |
| **Appointment kept** | 1. YES 2. NO |
| **INH Received** | 1. YES 2. NO |
| **Adherence score** | 1. Good (>95%) 2. Fair (85-95%) 3. Poor (<85%) |
| **Reasons for poor adherence** | 1. Side effects 2. Illness/hospital 3. Drugs stock-outs  4. Patient lack finances 5. Other patient’s reason |
| **TB status** | 1. No signs 2. Presumptive TB case  3. Diagnosed with TB 4. On TB treatment |
| **Weight (Kgs)** | _______________________________________________ |
| **Side effects** | 1. YES 2. NO |
| **Specify side effects** | 1. Numbness, tingling & burning sensation  2. Blurred or loss of vision 3. Convulsion  4. Mood change 5. Unusual bleeding 6. Skin rash  7. Sore throat 8. Joint pain 9. Fever 10. Dizzyness  11. Loss of appetite, nausea & vomiting 12. Dark urine  13. Other (specify)__________________ |

| **End of 3^rd^ month of therapy** | |
| --- | --- |
| **Visit date (day/month)** | ______/____________ |
| **Appointment kept** | 1. YES 2. NO |
| **INH Received** | 1. YES 2. NO |
| **Adherence score** | 1. Good (>95%) 2. Fair (85-95%) 3. Poor (<85%) |
| **Reasons for poor adherence** | 1. Side effects 2. Illness/hospital 3. Drugs stock-outs  4. Patient lack finances 5. Other patient’s reason |
| **TB status** | 1. No signs 2. Presumptive TB case  3. Diagnosed with TB 4. On TB treatment |
| **Weight (Kgs)** | _______________________________________________ |
| **Side effects** | 1. YES 2. NO |
| **Specify side effects** | 1. Numbness, tingling & burning sensation  2. Blurred or loss of vision 3. Convulsion  4. Mood change 5. Unusual bleeding 6. Skin rash  7. Sore throat 8. Joint pain 9. Fever 10. Dizziness  11. Loss of appetite, nausea & vomiting 12. Dark urine  13. Other (specify) |
| **End of 4^th^ month of therapy** | |
| **Visit date (day/month)** | ______/____________ |
| **Appointment kept** | 1. YES 2. NO |
| **INH Received** | 1. YES 2. NO |
| **Adherence score** | 1. Good (>95%) 2. Fair (85-95%) 3. Poor (<85%) |
| **Reasons for poor adherence** | 1. Side effects 2. Illness/hospital 3. Drugs stock-outs  4. Patient lack finances 5. Other patient’s reason |
| **TB status** | 1. No signs 2. Presumptive TB case  3. Diagnosed with TB 4. On TB treatment |
| **Weight (Kgs)** | _______________________________________________ |
| **Side effects** | 1. YES 2. NO |
| **Specify side effects** | 1. Numbness, tingling & burning sensation  2. Blurred or loss of vision 3. Convulsion  4. Mood change 5. Unusual bleeding 6. Skin rash  7. Sore throat 8. Joint pain 9. Fever 10. Dizziness  11. Loss of appetite, nausea & vomiting 12. Dark urine  13. Other (specify)_____________ |

| **End of 5^th^ month of therapy** | |
| --- | --- |
| **Visit date (day/month)** | ______/____________ |
| **Appointment kept** | 1. YES 2. NO |
| **INH Received** | 1. YES 2. NO |
| **Adherence score** | 1. Good (>95%) 2. Fair (85-95%) 3. Poor (<85%) |
| **Reasons for poor adherence** | 1. Side effects 2. Illness/hospital 3. Drugs stock-outs  4. Patient lack finances 5. Other patient’s reason |
| **TB status** | 1. No signs 2. Presumptive TB case  3. Diagnosed with TB 4. On TB treatment |
| **Weight (Kgs)** | _______________________________________________ |
| **Side effects** | 1. YES 2. NO |
| **Specify side effects** | 1. Numbness, tingling & burning sensation  2. Blurred or loss of vision 3. Convulsion  4. Mood change 5. Unusual bleeding 6. Skin rash  7. Sore throat 8. Joint pain 9. Fever 10. Dizziness  11. Loss of appetite, nausea & vomiting 12. Dark urine  13. Other (specify) |
| **End of 6^th^ month of therapy** | |
| **Visit date (day/month)** | ______/____________ |
| **Appointment kept** | 1. YES 2. NO |
| **INH Received** | 1. YES 2. NO |
| **Adherence score** | 1. Good (>95%) 2. Fair (85-95%) 3. Poor (<85%) |
| **Reasons for poor adherence** | 1. Side effects 2. Illness/hospital 3. Drugs stock-outs  4. Patient lack finances 5. Other patient’s reason |
| **TB status** | 1. No signs 2. Presumptive TB case  3. Diagnosed with TB 4. On TB treatment |
| **Weight (Kgs)** | _______________________________________________ |
| **Side effects** | 1. YES 2. NO |
| **Specify side effects** | 1. Numbness, tingling & burning sensation  2. Blurred or loss of vision 3. Convulsion  4. Mood change 5. Unusual bleeding 6. Skin rash  7. Sore throat 8. Joint pain 9. Fever 10. Dizziness  11. Loss of appetite, nausea & vomiting 12. Dark urine  13. Other (specify)__________________ |

| **IPT Treatment Outcomes** | |
| --- | --- |
| **Treatment outcomes** | 1. Completed 2. Died 3. LTFU 4. Stopped |
| **Reasons for stopping** | 1. Side effects 2. Treatment interruption  3.Developed active TB 4.Others (specify) |

**FORM 2: TB INFORMATION**

**HMIS FORM 096a: HEALTH UNIT TB REGISTER**

| **Patient’s baseline characteristics at TB diagnosis/TB treatment initiation** | |
| --- | --- |
| **Health facility details** |  |
| Health facility | 1. Kiswa HC III  2. Kawaala HC III  3. Komamboga HC III  4. Kitebi HC III  5. Kisugu HC III  6. Kisenyi HC IV |
| **Patient’s details** | |
| Sex | 1. Male 2. Female 3. Not specified |
| DOB (birthdate) DD/MM/YYY | ______________ |
| Age (years) at TB treatment start | _____________ |
| **Address** |  |
| District | _________________________________________ |
| Division/Sub-county | _________________________________________ |
| Parish/ward | _________________________________________ |
| Village/Zone/Cell | _________________________________________ |
| **TB diagnosis information** | |
| TB diagnosis date (dd/mm/yyyy) |  |
| Place of TB diagnosis | 1. OPD 2. ANC 3.TB clinic 4.HIV clinic 5. Community 6.Other specify |
| Disease class (ie. TB diagnosis type) | 1. Bacteriologically confirmed PTB (P-BC)  2. Clinically Diagnosed PTB (P-CD)  3. Extra-Pulmonary TB (EP)  Others, specify ___________________________________  ___________________________________________ |
| Type of patient | 1. New (N)  2. Relapse (R)  3. Failure (F)  4. Loss to follow-up (L)  Others specify _______________________________  ___________________________________________ |
| Date of TB treatment start (dd/mm/yyyy) | ____/____/________ |
| HIV test results | 1. Positive (CT1)  2. Negative (CT2)  3. Unknown/Undocumented |
| ART status | 1.Yes 2.No 3.Unknown |
| ARTstartdate (dd/mm/yyyy) | ____/____/________ |

**HMIS FORM 089: TB LABORATORY REGISTER**

| **Description** | **Response** |
| --- | --- |
| Laboratory name |  |
| 1. Lab serial no |  |
| 2. Date (DD/MM/YYYY) |  |
| 3. Name | __________________________________ |
| 5. Sex (M/F) | 1. Male 2. Female |
| 6. Age (years) |  |
| 7. Address: | County: |
|  | Sub-county: |
|  | Parish: |
|  | Village (LC1): |
| 7. Name of treatment unit |  |
| 8. Patient number |  |
| 8a. Presumptive TB/Unit no. |  |
| 8b. District TB no. |  |
| 9. Examination type | 1. Pre-treatment Diagnosis |
|  | 2. Follow-up: Month 1 2 3 4 5 6 ___________ |
| Examination results |  |
| Smear results | **Date (DD/MM/YYYY)**: |
|  | **Smear results**:  0. 0 = no AFB  1. (1-9) = exact number if 1-9 AFB/100 HPF (scanty)  2. + = 10-99 AFB/100 HPF  3. ++ = 1-10 AFB/HPF  4. +++ = >10 AFB/HPF |
| NAAT eg. Gene Xpert | **Date (DD/MM/YYYY)**: |
|  | **GeneXpert Results**:  1. T = MTB detected, rifampicin resistance not detected  2. RR = MTB detected, rifampicin resistance detected  3. TI = MTB detected, rifampicin resistance indeterminate 4. N = MTB not detected  5. I = indeterminate(invalid / no result / error) |
| 10. HIV status | 1. **CT1**= positive results;  2. **CT2**= Negative results |

**FORM 3: PREGNANCY/MATERNITY DATA**

**HMIS FORM 071: INTERGRATED ANTENETAL REGISTER**

***Form should be completed separately for every ANC visit)***

| **DESCRIPTIONS** | **RESPONSES** |
| --- | --- |
| **2. Client no.** |  |
| **3. Client name** (surname and first name in full) | Surname:____________________ |
|  | First name:_____________________ |
| **4. Village + Parish** (where the client resides) | Village: ____________________________ |
|  | Parish: ____________________________ |
| **5. Phone no.** | **____________________________** |
| **6. Age** (complete years) | **_______________________ years** |
| **Age groups (years)** | **1.** 10 – 19, **2.** 20 – 25, **3.** >=25 |
| **7. ANC visit**  [Tick the ANC visit for this visit] | **1^st^ 2^nd^ 3^rd^ 4^th^ 5^th^ 6^th^ 7^th^ 8^th^ ________** |
| **8. Gravida** (pregnancy in sequence) |  |
| **Para** (pregnancies carried beyond 7 months that the client has had before (exclude the current pregnancy) |  |
| **9a. Gestation age (**current ANC visitdate – LNMP date) **in weeks** |  |
| **9b. ANC1 timing** | During 1^st^ trimester (<=14 gest week)  After 1^st^ trimester (>14 gest week) |
| **10. Expected Date of Delivery (EDD)**  EDD = LNMP date + 9 months & 7 days | (DD/MM/YYYY):________________ |
| **11. Weight, Height , MUAC** | Weight (kg) : ________________ |
|  | Height (cm) : ________________ |
|  | MUAC (cm) : ________________ |
| **12. Blood pressure** |  |
| **13. eMTCT (see codes in this table footnote)** | **Woman (W) _________________** |
|  | **Partner (P) __________________** |
| **14a. Diagnosis** (findings after clinical assessment) | 1. Normal Pregnancy (NP),  2. Malaria, High blood pressure  3. Obstructed labour  4. Puerperal sepsis  5. Sepsis related to pregnancy  6. Anaemia in pregnancy  7. Ruptured uterus  8. Ectopic pregnancy  9. Other Complications of pregnancy (specify) |
| **14b. WHO CLINICAL STAGE/ CD4/ VIRAL LOAD**  If the pregnant mother is HIV positive.  Add test date eg. CD4 350 12/03/2013, Stage 1. | **WHO clinical stage:_____________** |
|  | **CD4 count: _____________ Date: ____________** |
|  | **Viral load: ______________ Date: ____________** |
| **15. ARV drugs** | 1. **ART**: Initiated ART for eMTCT on that visit  2. **ARTK**: Client already on HAART |
| **Pre-ART no: (IDCNO???)** |  |
| **17. TB status:** | 1. **No signs** = no signs or symptoms of TB  2. **Suspect** = TB refer or sputum sent  3. **TB Diagnosed** = Diagnosed with TB  4. **TB Rx** = currently on TB treatment |
| **18. Haemoglobin (g/dl)** |  |
| **19. Syphilis test result (Syp W/P):** | **1. Rx:** Client tested and results given - client reactive and given treatment  **2. +ve:** Client tested positive but not yet given treatment  **3. NR:** Client tested results given- client non-reactive  **4. NT:** Client not tested for syphilis |
| **22. IPT (Intermittent Preventive Treatment against malaria) Dose** | 1. Fansidar  2. Septrin (CTX) |
| **24. MEBENDAZOLE DOSE:** | **1. Tick** (**√**) – if received  2. **X** – if not yet received yet she’s due  3. **C** – if she completed |
| **25. IRON supplement** | 1. Yes - a tick (√) if a woman has received iron  2. No - |
| **FOLIC ACID** | 1. Yes - a tick (√) if a woman has received iron  2. No - |
| **26. Other treatments (Specify)** | **______________________________________** |
| **27. Referral In/Out:** | **1. REF OUT - referred out of the facility**  **2. REF IN** - referred into this health unit from another site |
| **28. Complications / Risk factors (specify)** |  |

**(13) eMTCT CODES:**

**Codes for clients who are newly tested in ANC**

**C**  Counseled but declined HIV testing

**T**  Counseled and tested but didn’t receive results

**TR** Counseled tested and results given - Client tested HIV Negative

**TRR** Counseled tested and results given - client tested HIV Positive

**Codes for revisit clients who come to ANC with known status**

TR√ Revisit ANC clients, who were tested for HIV on a previous ANC visit with known HIV Negative status TRR√ Revisit ANC clients who were tested for HIV on a previous ANC visit with known HIV Positive status

**Codes for new clients who come to ANC with documented evidence of test results**

TRK *Clients who tested HIV Negative within 4 weeks prior to arrival in ANC

TRRK Clients who tested HIV Positive prior to arrival in ANC * Clients who tested HIV Negative within 4 weeks prior to arrival in ANC: If the test is negative and was done 4 weeks prior to the visit then a re-test is recommended. ANC retesting should be done in 2nd /3rd trimester

**Codes for clients who are re-tested later in pregnancy**

TR+ Clients who tested HIV Negative on a re-test

TRR+ Clients who tested HIV Positive on a re-test

If the initial HIV test is negative a re-test is recommended later during the pregnancy

**(15) ARV DRUGS codes**

If a HIV positive pregnant mother is started on ARVs on that visit, use the following codes to indicate which drug is prescribed. If the pregnant mother is HIV negative, write NA.

**ART** Initiated ART for eMTCT on that visit

**ARTK** Client already on HAART for their own health prior to the first ANC visit or before this pregnancy

If this is a revisit and the HIV positive pregnant mother is already on ARVs, write the code with a tick “√“

**ART√** - Client is a revisit and was prescribed on an earlier visit

**ARTK√** - Client is a revisit and was prescribed on an earlier visit even for those who were on ART before the pregnancy

**HMIS FORM 072: INTERGRATED MATERNITY REGISTER**

| **DESCRIPTIONS** | **RESPONSES** |
| --- | --- |
| **4. Name** | **________________________________-** |
| **5. Address:** | Village:_______________________ |
|  | Parish:_______________________ |
| **6. Phone no.** | ___________________________ |
| **7. Age** | In complete year _____ |
| **Age groups** | 1. 10 – 19 yrs 2. 20 – 24 yrs 3. ≥ 25 years |
| **8. Gravidity/Para** | Gravidity ___________- |
|  | Para ____________ |
| **9. Weeks of gestation** | __________________ |
| **10a. Final diagnosis** | 1. Transverse position  2. “BBA” born before arrival  3. Born at the time of discharge  4. Referral  5. Death |
| **Obstetrical complications experienced by the time of admission, or during the course of stay at the health unit** | 1. Abortions  2. APH  3. PPH  4. Malaria in pregnancy  5. High blood pressure in pregnancy  6. Obstructed labour  7. Puerperal sepsis  8. Sepsis related to pregnancy  9. Anaemia in pregnancy  10. Ruptured uterus  11. Ectopic pregnancy  12. Other Complications of pregnancy |
| **Pregnancy outcomes**  **Note:** *Missing pregnancy outcome should be obtained by phone call tracking. However, this should be specified***.** | 1. Live birth  2. Pre-term: early or very preterm birth delivery (before 32 weeks of gestation)  3. Miscarriage/Spontaneous abortion  4. Stillbirth  5. Other (specify) |
| **10b. WHO clinical stage/ CD4 /viral load** | WHO clinical stage: ______ |
|  | CD4 count: ___________ |
|  | Viral load: ___________ |
| **11. Mode of delivery** | 1. Normal Vaginal delivery  2. Vacuum extraction  3. Caesarean section |
| **Other management procedures (If not “Normal vaginal delivery)** | 1. Manual Vacuum Aspiration (MVA)  2. Dilatation and Curettage (D & C)  3. Laparotomy  4. Hysterectom |
| **12. Date of delivery (DD/MM/YYYY)** | _____________________ |
| **13. Time of delivery** | _________________ AM/PM |
| **14. Management of 3^rd^ stage** | 1. Ergometrine  2. Pitocin  3. Misoprostol |
| **15. Other treatment given** | **_____________________________** |
|  | **____________________________** |
| **CHILD INFORMATION** | |
| **19. Apgar Score** |  |
| **20. Sex** | 1. Male  2. Female |
| **21. Not breathing at birth** | **1. SS -** baby breathes after Stimulation and or Suction procedure  **2. BM -** baby breathes after Bag and Mask procedure  **3. BMD -** baby dies after Bag and Mask procedure |
| **26. Weight (grams)** | **__________________________** |
| **DISCHARGE INFORMATION** | |
| **30. Condition Of Mother at Discharge** | 1. D - Woman discharged is alive  2. DD - Woman died  3. R - If the woman run away or left before being discharged  4. T-Woman transferred to another facility  5. DF-Woman discharged alive with Fistula  6. DDF-Woman died with Fistula  7. RF-Woman ran away with Fistula  8. TF-Woman transferred with Fistula  9. Others (specify)_________________ |
| **31. Condition of baby at discharge**  (*Please note that babies older than 1day are captured in postnatal register*) | 1. MSB - Macerated still birth  2. FSB - Fresh still birth  3. NND - Immediate Neonatal Death (death within 24hrs)  4. AL - Live Baby BDF - Baby born with defect  Specify the defect ____________________________ |
| **32. DELIVERED BY:** | Midwife  Doctor  Others (specify)_______________________ |
| **33. PNC at 6H (***Record the date when the mother/baby attended postnatal care (PNC) if the attendance was within six hours after delivery***)** | Yes  No  If Yes; Date: _______________ |
| **34. Date of Discharge** | **_________________________** |
|  |  |

**(10a) FINAL DIAGNOSIS: This refers to Obstetrical diagnosis, such as “transverse position”, “BBA” born before arrival, at the time of discharge or referral or death. For HIV positive mothers note the WHO clinical stage or CD4 test results. If there are obstetrical complications experienced by the time of admission, or during the course of stay at the health unit, they should be also entered and coded as follows: 1. Abortions 2. APH 3. PPH 4. Malaria in pregnancy 5. High blood pressure in pregnancy 6. Obstructed labour 7. Puerperal sepsis 8. Sepsis related to pregnancy 9. Anaemia in pregnancy 10. Ruptured uterus 11. Ectopic pregnancy 12. Other Complications of pregnancy**

**Definition: An antepartum haemorrhage (APH) is bleeding from the vagina that occurs after the 20th week of pregnancy and before the birth of your baby. The common causes of bleeding during pregnancy are cervical ectropion, vaginal infection, placental edge bleed, placenta praevia or placental abruption. Mar 16, 2018.**

**Postpartum bleeding or postpartum hemorrhage (PPH) is often defined as the loss of more than 500 ml or 1,000 ml of blood within the first 24 hours following childbirth. Some have added the requirement that there also be signs or symptoms of low blood volume for the condition to exist**

**(9) WEEKS OF GESTATION: The age of the pregnancy in weeks is entered in this column, e.g. 34 to mean 34 completed weeks of gestation. Indicate if the delivery is term (T) or pre-term (P) i.e. less than 37 weeks of gestation**

**DELIVERY INFORMATION:**

**(11) MODE OF DELIVERY/MANAGEMENT PROCEDURE: The techniques used during delivery, such as,”Normal Vaginal delivery”, “vacuum extraction “, “caesarean section”, etc. If other management procedures were done to manage the condition, they are entered here, such as Manual Vacuum Aspiration (MVA), Dilatation and Curettage (D & C), laparotomy, hysterectomy, etc.**

**(12) DATE OF DELIVERY: The date when the mother delivers is recorded in this column**

**(13) TIME OF DELIVERY: The time (in 12 hours) when the mother delivers is indicated in this column.**

**(14) MANAGEMENT OF 3RD STAGE OF LABOUR: Note what the mother was given, codes used for description are: 1. Ergometrine 2. Pitocin 3. Misoprostol**

**(15) OTHER TREATMENT GIVEN Medicines given are entered under this column, such as Misoprostol, Magnesium sulfate, Antibiotics, IVfluids, Blood transfusion, Injection Dexamethasone during preterm labor etc**

**(19) Apgar score** is a test given to newborns soon after birth. This test checks a baby's heart rate, muscle tone, and other signs to see if extra medical care or emergency care is needed. The test is usually given twice: once at 1 minute after birth, and again at 5 minutes after birth.

**The Apgar score is based on a total score of 1 to 10. The higher the score, the better the baby is doing after birth. A score of 7, 8, or 9 is normal and is a sign that the newborn is in good health.Oct 11, 2018**

**HMIS FORM 078: INTERGRATED POSTNATAL REGISTER**

| **DESCRIPTIONS** | **RESPONSES** |
| --- | --- |
| **Mother’s information** |  |
| **2. Client no. (number ‘‘001/YEAR’’ on the first clinic day in July each year)** |  |
| **3. Mother name** | **________________________________-** |
| **5. Address:** | Village:_______________________ |
|  | Parish:_______________________ |
| **6. Phone no.** | ___________________________ |
| **7. Age** | In complete year _____ |
| **Age groups** | 1. 10 – 19 yrs 2. 20 – 24 yrs 3. ≥ 25 years |
| **8. Timing for PNC visit** | 1. 6hrs 2. 6Days 3. 6weeks 4. 6months |
| **12. Weight (kgs)** | Para ____________ |
| **MUAC** |  |
| **16a. Diagnosis** |  |
| **16b. WHO/ CLINICAL STAGE/ CD4/ VIRAL LOAD** | **WHO clinical stage:_____________** |
|  | **CD4 count: _____________ Date: ____________** |
|  | **Viral load: ______________ Date: ____________** |
| **Baby’s information** |  |
| **18. Status of baby** | 1. **AL** = baby is alive  2. **NND7**= dead within the first seven (7) days  3. **NND28** = dead between eight (8) and twenty-eight (28) days  4. Other (specify)______________________ |
| **19. Baby age (weeks)** |  |
| **20. Baby’s weight** |  |
| **21. Baby’s diagnosis** | 1. Normal – if the child has no infection  2. Pneumonia,  3. Malaria  4. Other (specify)_________________  ________________________________ |
| **22. Infant feeding options** | **1. EBF** – For Exclusive breast feeding  **2. RF** – Replacement Feeding  **3. MF** – Mixed Feeding  **4**. Others (specify) |
| **24. Other treatment** |  |
| **25. Referred to where** |  |
| **Referred from where** |  |

**FORM 4: EXPOSED INFANT**

**HMIS FORM 082a: EXPOSED INFANT CLINICAL CHART**

| **General clinic information** | |
| --- | --- |
| **Health facility** |  |
| **District** |  |
| **Exposed infant information** | |
| **EI Number** |  |
| **Date Chart opened** |  |
| **Infant name** |  |
| **Entry point (Clinic/Ward)** |  |
| **Sex** | 1. Male 2. Female |
| **Date of birth (DD/MM/YYYY)** |  |
| **Age (month)** |  |
| **Date of NVP start** |  |
| **Date of CTX start** |  |
| **MOTHER’S/CARE GIVER’S FOLLOW-UP INFORMATION** | |
| **Mother’s name** |  |
| **Common name** |  |
| **District** |  |
| **Country** |  |
| **Sub county** |  |
| **Parish** |  |
| **Village** |  |
| **Child’s HIV testing information** | |
| **1^st^ PCR (date/result)** | Date(dd/mm/yyyy): ________ Result: 1.POS 2.NEG |
| **2^nd^ PCR (date/result)** | Date(dd/mm/yyyy): ________ Result: 1.POS 2.NEG |
| **Repeat PCR (date/result)** | Date(dd/mm/yyyy): ________ Result: 1.POS 2.NEG |
| **18 months rapid test (date/result)** | Date(dd/mm/yyyy): ________ Result: 1.POS 2.NEG |
| **Final HIV status (date/result)** | Date(dd/mm/yyyy): ________ Result: 1.POS 2.NEG |

******************* E N D *********************
