# Supplementary material for: Increased uptake of tuberculosis preventive therapy (TPT) among people living with HIV following the 100-days accelerated campaign: A retrospective review of routinely collected data at six urban public health facilities in Uganda
Source: PLoS One. 2023 Feb 23;18(2):e0268935. doi: 10.1371/journal.pone.0268935 (PMC9949662; doi:10.1371/journal.pone.0268935)
Supplement: Attachment — Submitted filename: Comments_TPT.pdf [file pone.0268935.s004.pdf]

Increased uptake of tuberculosis preventive therapy (TPT) among people living with HIV following the 100-days accelerated campaign: A retrospective review of routinely collected data at six urban public health facilities in Uganda

Musaazi, J et al

Generally, this is an important manuscript touching on a topical issue- tuberculosis preventive therapy (TPT). I found it generally well written. However, I have a few concerns as listed below.

Comments

Abstract

The Uganda Ministry of Health launched a 100-day campaign to scale-up TPT in PLHIV in July 2019.

Aim: We sought to examine the effect of the campaign on trends of TPT uptake and characteristics associated with TPT uptake and TPT completion among persons in HIV care. We retrospectively reviewed routinely collected data from 2016 to 2019 at six urban public health facilities in Uganda.

Comment 1

If the launch of the campaign was July 2019, and the study reviewed records from 2016 to 2019 at six urban public health facilities in Uganda. <u>Wasn't it too early to assess the effect of the campaign?</u>

Results

On average a total of 43,215 patients aged 15 years and above were eligible for TPT each 60 calendar year at the six health facilities. More than 70% were females and median age was 34 years 61 (inter-quartile range 28 to 41 years on overall).

Comment 2

This statement is confusing 'On average a total of 43,215 patients aged 15 years and above were eligible for TPT each 60 calendar year at the six health facilities.' So the 70% females is of the total eligible patients for all the four years? What was the total eligible patients in the 4 years?

Introduction

Tuberculosis (TB) is the most frequent cause of Acquired Immunodeficiency Syndrome (AIDS)-related deaths worldwide despite the wide availability of antiretroviral therapy (ART)(1). Tuberculosis preventive treatment (TPT) reduces the risk of developing active TB(2) and TB-associated mortality.

Comment 3

In line 78, tuberculosis is abbreviated as 'TB' and so subsequently the authors should use the abbreviation 'TB' throughout the document eg the beginning of **line 79**.

Line 102-106 One of the interventions done by the Uganda MoH was the 100-day accelerated isoniazid preventive therapy (IPT) scale-up campaign launched on 3rd 103 July 2019(16). This campaign aimed to enroll 300,000 PLHIV on isoniazid preventive therapy at 1947 ART sites by 30th September 2019. There is scanty published data showing the trends of prevalence of TPT uptake and

completion, and the impact of the 2019 Ugandan MoH 100-day TPT scale-up campaign on these trends.

Comment 4

Give the barriers mentioned i.e. 'inadequate TPT supply in health facilities, frequent drug stock-outs, poor patient adherence, limited TPT knowledge by health workers, lack of confidence in symptom-based TB screening alone, and fear of isoniazid resistance,' can the authors describe what exactly the entailed this campaign of 100 days to be able to acheive these very impressive results in a very short time July to December, 2022. Considering that the first three months (100 days) could have been invested in the campaign leaving only 3months for implementation and evaluation (including uptake and completion). This means that the time to assess the impact was not long enough and probably these results could be due to some other factors and not necessarily the campaign! The methods section should have this section explaining the details of the campaign/ catch up strategy!

Meanwhile data collection was done in Data were collected from July 2020 to March 2021. (Line 160-161), just wondering why then did evaluate the campaign, a year after its launch, to be able take care of all effects.

Discussion

Comment 5

Line 333-336. Although it was impossible to explore reasons for poor TPT uptake given the retrospective nature of our study, some of the reasons cited for low TPT uptake highlighted in Kalema et.al study included: limited capacity of clinicians to exclude TB using symptoms alone, fear of promoting drug resistance due to isoniazid monotherapy and inconsistent TPT drug supplies(17). Can the authors explain how these barriers were overcame by the 100-days campaign!

Conclusion

Comment 6

Some of the issue raised in the conclusion are not supported by this study's findings, for example;). **Line 373-374**. 'Also, there is need for constant refresher trainings for health workers to understand importance of 374 TPT, and consistent TPT supply at health facilities.'

Need to recognize the limitation of short time period to evaluate the campaign.
