# Supplementary material for: Increased uptake of tuberculosis preventive therapy (TPT) among people living with HIV following the 100-days accelerated campaign: A retrospective review of routinely collected data at six urban public health facilities in Uganda
Source: PLoS One. 2023 Feb 23;18(2):e0268935. doi: 10.1371/journal.pone.0268935 (PMC9949662; doi:10.1371/journal.pone.0268935)
Supplement: Attachment — Submitted filename: Response to Reviewers.pdf [file pone.0268935.s005.pdf]

The Editor,
PLoS One

Date: 28th July 2022

Dear Sir,

RE: RESPONSE TO REVIEWERS' COMMENTS MANUSCRIPT ID: PONE-D-22-13680 "Increased uptake of tuberculosis preventive therapy (TPT) among people living with HIV following the 100-days accelerated campaign: A retrospective review of routinely collected data at six urban public health facilities in Uganda".

The authors of the reviewed manuscript thank the academic editor and reviewers for their consideration and opinions on the manuscript. We particularly thank the reviewers for their careful review of the manuscript and the useful comments. Below, we provide detailed point-by-point responses to the reviewers' comments in italics and underlined. We have also tracked the changes in the revised manuscript.

ACADEMIC EDITORS' COMMENT

1: Please ensure that your manuscript meets PLOS ONE's style requirements, including those for file naming.

Response: *The revised manuscript has been reformatted according to PLoS One's style requirements.*

2: Thank you for stating the following in the Funding Section of your manuscript:

"Support for data collection was provided by EDCTP, grant number: 1) EDCTP-RegNET2015-1104, and 2) Fogarty International Center, National Institutes of Health (grant # 2D43TW009771-06 "HIV and co-infections in Uganda"

We note that you have provided funding information that is not currently declared in your Funding Statement. However, funding information should not appear in the Acknowledgments section or other areas of your manuscript. We will only publish funding information present in the Funding Statement section of the online submission form.

Please remove any funding-related text from the manuscript and let us know how you would like to update your Funding Statement. Currently, your Funding Statement reads as follows:

"Support for data collection was provided by EDCTP TB NODE, grant number: 1) EDCTP-RegNET2015-1104, and 2) Fogarty International Center, National Institutes of Health (grant # 2D43TW009771-06 "HIV and co-infections in Uganda."

The funders had no role in the study design, data collection, and analysis, decision to publish, or preparation of the manuscript."

Please include your amended statements within your cover letter; we will change the online submission form on your behalf.

***Response:** Thank you for the comment. The funding-related text has been removed from the manuscript.*

The funding statement remains as, "Support for data collection was provided by EDCTP TB NODE, grant number: 1) EDCTP-RegNET2015-1104, and 2) Fogarty International Center, National Institutes of Health (grant # 2D43TW009771-06 "HIV and co-infections in Uganda."

It was also stated in the online initial submission.

3. We note that you have indicated that data from this study are available upon request. PLOS only allows data to be available upon request if there are legal or ethical restrictions on sharing data publicly. For more information on unacceptable data access restrictions, please see http://journals.plos.org/plosone/s/data-availability#loc-unacceptable-data-access-restrictions.

In your revised cover letter, please address the following prompts:

a) If there are ethical or legal restrictions on sharing a de-identified data set, please explain them in detail (e.g., data contain potentially sensitive information, data are owned by a third-party organization, etc.) and who has imposed them (e.g., an ethics committee). Please also provide contact information for a data access committee, ethics committee, or other institutional body to which data requests may be sent.

b) If there are no restrictions, please upload the minimal anonymized data set necessary to replicate your study findings as either Supporting Information files or to a stable, public repository and provide us with the relevant URLs, DOIs, or accession numbers. For a list of acceptable repositories, please see http://journals.plos.org/plosone/s/data-availability#loc-recommended-repositories.

We will update your Data Availability statement on your behalf to reflect the information you provide.

***Response:** Thank you for the guidance.*

There are no restrictions, and anonymized data set was uploaded at the initial submission.

4. Please upload a copy of Figures 2-5, to which you refer in your text on page 13 and 15. If the figure is no longer to be included as part of the submission please remove all reference to it within the text.

***Response:** The figures (2-5) have been uploaded.*

5. Please review your reference list to ensure that it is complete and correct. If you have cited papers that have been retracted, please include the rationale for doing so in the manuscript text, or remove these references and replace them with relevant current references. Any changes to the reference list should be mentioned in the rebuttal letter that accompanies your revised manuscript. If you need to cite a retracted article, indicate the article's retracted status in the References list and also include a citation and full reference for the retraction notice.

***Response:** The reference list has been reviewed for completeness and correctness.*

ADDITIONAL EDITOR COMMENTS:

Increased uptake of tuberculosis preventive therapy (TPT) among people living with HIV following the 100-days accelerated campaign: A retrospective review of routinely collected data at six urban public health facilities in Uganda

Musaazi, J et al

Generally, this is an important manuscript touching on a topical issue- tuberculosis preventive therapy (TPT). I found it generally well written. However, I have a few concerns as listed below.

Comments

Abstract

The Uganda Ministry of Health launched a 100-day campaign to scale-up TPT in PLHIV in July 2019. Aim: We sought to examine the effect of the campaign on trends of TPT uptake and characteristics associated with TPT uptake and TPT completion among persons in HIV care. We retrospectively reviewed routinely collected data from 2016 to 2019 at six urban public health facilities in Uganda.

Comment 1

If the launch of the campaign was July 2019, and the study reviewed records from 2016 to 2019 at six urban public health facilities in Uganda. Wasn't it too early to assess the effect of the campaign?

***Response:** We thank the reviewer for the comment.*

The main aim of the 100-day campaign was to initiate TPT for 300,000 adults and children living with HIV and under 5 years contacts of TB patients and ensure 100% completion rates (by 30th September 2019) of individuals that initiated 6-months TPT dose in quarters: October - December 2018, and January-February 2019.

We, therefore, believe that our assessment of the effect of this campaign at end of December 2019 was very timely and not too early.

However, a limitation of a short-time period evaluation of the campaign has also been added.

RESULTS

On average a total of 43,215 patients aged 15 years and above were eligible for TPT each 60 calendar year at the six health facilities. More than 70% were females and median age was 34 years 61 (inter-quartile range 28 to 41 years on overall).

Comment 2

This statement is confusing 'On average a total of 43,215 patients aged 15 years and above were eligible for TPT each 60 calendar year at the six health facilities.' So the 70% females is of the total eligible patients for all the four years? What was the total eligible patients in the 4 years?

Response: *We agree with the reviewer that the statement was a bit unclear. In the initial statement, we intended to provide a precise summary of the characteristics across the 4 years studied. Which we probably did not phrase very well. The statement has now been rephrased to read as below.*

On average a total of 43,215 patients aged 15 years and above were eligible for TPT each calendar year at the six health facilities. Across all the 4 years, more than 70% were females (range: 73.5% -74.6%) and median age ranged from 33 to 34 years."

Introduction

Tuberculosis (TB) is the most frequent cause of Acquired Immunodeficiency Syndrome (AIDS)-related deaths worldwide despite the wide availability of antiretroviral therapy (ART)(1). Tuberculosis preventive treatment (TPT) reduces the risk of developing active TB(2) and TBassociated mortality.

Comment 3

In line 78, tuberculosis is abbreviated as 'TB' and so subsequently the authors should use the abbreviation 'TB' throughout the document eg the beginning of **line 79**.

Response: *We thank the reviewer for the comment. This has been addressed.*

We have subsequently used the abbreviation for Tuberculosis "TB" throughout the document after the first occurrence in full name and abbreviation.

Line 102-106 One of the interventions done by the Uganda MoH was the 100-day accelerated isoniazid preventive therapy (IPT) scale-up campaign launched on 3rd 103 July 2019(16). This campaign aimed to enroll 300,000 PLHIV on isoniazid preventive therapy at 1947 ART sites by 30th September 2019. There is scanty published data showing the trends of prevalence of TPT uptake and completion, and the impact of the 2019 Ugandan MoH 100-day TPT scale-up campaign on these trends.

Comment 4

Give the barriers mentioned i.e. 'inadequate TPT supply in health facilities, frequent drug stock-outs, poor patient adherence, limited TPT knowledge by health workers, lack of confidence in symptom based TB screening alone, and fear of isoniazid resistance,' can the authors describe what exactly the entailed this campaign of 100 days to be able to achieve these very impressive results in a very short time July to December, 2022. Considering that the first three months (100 days) could have been invested in the campaign leaving only 3months for implementation and evaluation (including uptake and completion). This means that the time to assess the impact was not long enough and probably these results could be due to some other factors and not necessarily the campaign! The methods section should have this section explaining the details of the campaign/ catch up strategy! Meanwhile data collection was done in Data were collected from July 2020 to March 2021. (Line 160-161), just wondering why then did evaluate the campaign, a year after its launch, to be able take care of all effects.

Response: *We thank the reviewer for the comment.*

The actual implementation of rapid IPT initiation and enhanced IPT completion started immediately after the launch of the 100-day campaign. We also believe that during the period 3rd July 2019 to 30th September 2019, the 100-day accelerate IPT scale-up was the only intervention conducted in health facilities studied. The impressive impact of the campaign observed in our evaluation could be because the campaign addressed most of the initial barriers to TPT initiation and completion. For example, 1) through enhanced systems for TPT delivery, monitoring, and reporting outcomes, 2) mobilized a DHO-LED multi-stakeholder engagement for accelerated IPT implementation and increased accountability, e.t.c.

We had a statement in the first paragraph of the discussion section explaining the details of the campaign/catch-up strategy. However, we have further listed other activities that were done during the 100-day campaign that could have helped to achieve the success we observed.

Discussion

Comment 5

Line 333-336. Although it was impossible to explore reasons for poor TPT uptake given the retrospective nature of our study, some of the reasons cited for low TPT uptake highlighted in Kalema et.al study included: limited capacity of clinicians to exclude TB using symptoms alone, fear of promoting drug resistance due to isoniazid monotherapy and inconsistent TPT drug supplies(17). Can the authors explain how these barriers were overcame by the 100-days campaign!

Response: *Statements have been added on how barriers to TPT uptake and completion were addressed in the 100-day accelerated TPT scale-up campaign. Refer to paragraph #2 in the discussion section.*

Conclusion

Comment 6

Some of the issue raised in the conclusion are not supported by this study's findings, for example;). Line 373-374. 'Also, there is need for constant refresher trainings for health workers to understand importance of 374 TPT, and consistent TPT supply at health facilities.'

Response: *The statement has been removed.*

Need to recognize the limitation of short time period to evaluate the campaign.

Response: *A limitation of a short-time period evaluation of the campaign has been added.*

Thank you

Joseph Musaazi

Corresponding Author.
