# Supplementary material for: Increased uptake of tuberculosis preventive therapy (TPT) among people living with HIV following the 100-days accelerated campaign: A retrospective review of routinely collected data at six urban public health facilities in Uganda
Source: PLoS One. 2023 Feb 23;18(2):e0268935. doi: 10.1371/journal.pone.0268935 (PMC9949662; doi:10.1371/journal.pone.0268935)
Supplement: Attachment — Submitted filename: Response to Reviewers.docx [file pone.0268935.s006.docx]

The Editor,

PLoS One

Date: 24^th^ November 2022

Dear Sir,

**RE: RESPONSE TO REVIEWERS’ COMMENTS MANUSCRIPT ID: PONE-D-22-13680R1 “Increased uptake of tuberculosis preventive therapy (TPT) among people living with HIV following the 100-days accelerated campaign: A retrospective review of routinely collected data at six urban public health facilities in Uganda”.**

*The authors of the reviewed manuscript thank the academic editor and reviewers for their consideration and careful review of the manuscript. Below, we provide detailed point-by-point responses to the reviewers’ comments in italics and underlined. We have also tracked the changes in the revised manuscript.*

**ACADEMIC EDITORS’ COMMENT**

Please note that I was asked to step in as academic editor (invitation accepted on 13/09/2022) since the initial editor was no longer available. In the interest of rigorous peer review process, the manuscript was sent out to four independent external reviewers. They made important recommendations and the manuscript cannot be accepted before this a revision. Please carefully respond to the reviewers' comments in a detailed point-by-point reply (also included revised text/inserts), and pay particular attention to the following points:

1.	Revise methods, including study population and study design, data extraction (including statement on double entry) (see all Reviewers)

2.	Missing values (see also Reviewer 2): provide details according to STROBE statement, clearly present missing values

3.	Evaluation time 3 months after (Reviewer 2): explain and/or provide alternative analysis

4.	Revisit analyses and provide a more granular presentation of the data (by quarters, stratified by eligibility criteria for TPT, etc.), 2019 vs. all other years (see all 4 Reviewers)

5.	Clear description of the interventions (see Reviewer 1)

6.	Expand discussions (potential benefits and harms of such a campaign; see Reviewer 1)

7.	Clarify if other interventions took place and could be responsible for the increased uptake (see Reviewer 4)

**REVIEWERS' COMMENTS:**

**Reviewer #1:** Thank you for asking me to review this manuscript. Although data are interesting, there is currently insufficient methodological and reporting detail in the manuscript to assess what actually happened. I note that in the previous round of reviews, the Editor has already asked for much of this information to be provided. I have provided detailed comments below, and hope the authors are able to fully address them.

1. Lines 79-81 describe the Uganda programme approach to TPT. However, under these guidelines (as described), all PLHIV would take TPT intermittently for the remainder of their lives. Are there time-based criteria for initiation/reinitiating? In practice, what clinical event triggers initiation/reinitiation of TPT (HIV diagnosis/ART initiation etc)? A more careful description in the Methods of the programmatic approach as implemented, beyond what is listed in the guidelines, would be helpful to understand the approach to TPT provision.

***Response:*** *Thank you for the comment.*

*According to Ugandan HIV/TB guidelines TPT is only provided to all eligible individuals once in a lifetime and not intermittently. Once the TPT course is completed, the patient does not require another dose. Currently TPT is given to all new PLHIV and anyone in HIV care who never completed or interrupted a course of TPT. Also, patients on ART refill visits are always re-evaluated for TPT history and screened for TB before they are initiated on TPT.*

*We have edited the statement in the introduction section that highlights Uganda’s ministry of health TPT programme, and indicated that TPT is offered as a one course of treatment (see page 5, lines 77-79). Also, under setting in the methods section, we have further indicated that during the period studied, Isoniazid monotherapy for six months was the only TPT option available (see page 7, lines 118-120).*

*We have added more statements in the methods section (see page 7, lines 126 – 130 and page 9, lines 171-178) to highlight the programmatic approach as implemented, beyond what is listed in the guidelines. For example, in programmatic setting, depending on the stability of the patient in terms of health status and ART adherence, ART medications can be provided for either a month, or 3 months or 6 months. Therefore, TPT refill visits are aligned with ART refill visits in order to decrease the number of patients visits to the clinic which can be costly to the patient in terms of transport and time.*

2. Methods: add text to provide rationale for the selection of the 6 study HIV clinics, with particular attention to that characteristics of these clinics that mean that data can be generalised from them to the remainder of clinics in the country, and regionally.

***Response:*** *We have clarified the rationale for selection of the 6 study HIV clinics and briefly described the characteristics of these clinics (see pages 6, lines 103-116).*

*About generalisability, since Uganda is a limited-resource country and has a high burden of HIV and TB coinfections which is more pronounced in urban areas like Kampala than in rural areas, our findings can be generalisable to other similar resource-limited settings with high burden of TB and HIV coinfections. We included this under discussion section (see page 23, lines 438-439).*

3. Lines 106-111: Describe which body in Uganda has responsibility for purchasing, distribution and monitoring of TPT (HIV Programme, TB Programme, other, mixture?)

***Response:*** *In Uganda, TPT is procured under the Quantification planning and procurement Unit (QPPU) of the ministry of health. Distribution of TPT is done by the government national warehouse (National medical stores, i.e. NMS) while monitoring and support supervision is done by Ministry of Health under the National TB and leprosy program (NTLP).*

*The above statement has been added under setting in methods section (see page 7, lines 130-133)*

4. Lines 113-115: Inclusion and exclusion criteria are insufficiently detailed to allow readers to tell who results are applicable to. For example, as currently written, was an HIV-positive person taking ART for 15 years eligible to initiate IPT before the campaign, and during the campaign (to take a somewhat extreme example). If the eligibility criteria for initiation were substantially wider during the campaign, then of course more people are going to be “available” to be initiated onto IPT. However, in this case, the conclusion would be that a campaign is not needed, rather programmes should focus on changing eligibility criteria to boost numbers. Please clearer description of what actually happened before and during the campaign to allow readers to understand what the drivers of success were.

***Response:*** *The same national TPT guidelines were followed before and after the accelerated IPT campaign: All people living with HIV were eligible after excluding active TB disease, regardless of their duration on ART. However, during the 100-days campaign in 2019, the Uganda Ministry of Health aimed at achieving a rapid IPT initiation and completion during that period. Besides, the Ministry wanted to jump-start the TPT program and also address some challenges in the country TPT program e.g., limited isoniazid availability at health facilities due to supply chain challenges, lack of awareness of the potential benefits of isoniazid; health facilities, district & implementing partners not being held accountable for IPT performance.*

*In order to make it clear to the reader what happened before and during the campaign, we have added statements under setting in methods section describing the activities conducted during the 100-day accelerated IPT campaign (see page 8, lines 134-146).*

*We further provide the reference for more details about this campaign, i.e. reference #18: Uganda Ministry of Health. 100-Day Accelerated Isoniazid Preventive Therapy Scale Up Plan, 2019.*

5. Further to point above, is there any evidence to suggest that repeated/continuous courses of IPT are beneficial and not harmful to PLHIV? From figure 1, seems like people more than likely had more than one course of IPT. Was this supported by the national or WHO guidelines. Otherwise, I guess a programme wanting to increase “IPT delivery” could just repeatedly provide IPT to the same set of easier to reach people.

***Response:*** *Thank you for pointing out the need to clarify this point. There is no recommendation in place for repeated TPT. According to the Uganda Ministry of Health TPT guideline that applied during the period when the data was collected, people living with HIV who had never previously received IPT and did not have evidence of active TB received IPT.*

*Figure 1 has a footnote showing the number of patients who were excluded from the analysis in a specific year because of IPT history. We excluded both newly enrolled in the clinic with prior history of IPT uptake and the continuing patients initiated on IPT in the previous year.*

*To make this clearer, we have added a statement under the eligibility section indicating “Individuals who were initiated on TPT in the previous calendar year were excluded in the analysis of current year.” (see page 8, lines 153-157).*

6. Lines 117-124: Similar to points above - need to provide a detailed description of the procedures for assessment and initiation of TPT, not just what happened after initiation. The whole point of this study is to investigate whether a “campaign” increased TPT initiation, but we have essentially no information about what happened before the campaign, or during the campaign. I note that this information was previously asked for in the previous round of reviews, but has again not been provided here.

***Response:*** *Thank you for the comment.*

*The Uganda Ministry of Health has recommended TPT since 2006 under the national policy guidelines for TB/HIV collaborative activities [14,] but due to challenges with isoniazid stocks (because TB treatment is in fixed dose combinations), the number of people receiving TPT is limited to when a full six month course can be supplied. During the 100 days campaign, a significant stock of TPT was received and pushed to the facilities by National Medical Stores to initiate all eligible PLHIV. Prior to initiation of TPT, PLHIV were first screened for signs and symptoms of TB using the TB case finding form and any presumptive case would be first investigated to rule out TB before TPT initiation; those without TB symptoms would then be screened further to rule out any contraindication to isoniazid prior initiation to TPT (see S2 table X2 in supplementary 2, the checklists for screening for contraindications).*

*The above statement has been added under treatment procedure section in methods (see page 9, lines 165-181 and page 10 lines 182-184).*

7. Line 117: which “patients”?

***Response:*** *Thank you for the comment.*

*We have corrected the statement to indicate that “PLHIV” (see page 9, lines 165-167).*

8. Lines 126- needs much more information about the data extraction from paper records, and associated extraction from electronic pharmacy records. What data were extracted? How was quality of extraction assured? How was record linkage between paper and electronic records done and quality assured? How was missing data handled? Also needs key outcome definitions to be provided here (i.e. how was “TPT initiation” defined?)

***Response:*** *Thank you for the comment.*

*Responding to each individual question in this comment.*

*What data were extracted?*

*A list of data variables has been added under methods section in the data extraction and collection (see page10, lines 191-197). Also, data collection tool is attached as a separate supplementary material.*

*How was quality of extraction assured?*

*Under methods in the data extraction and collection section, we have added a statement that explains how data quality was assured as follows: “A structured questionnaire for extraction of TPT information from the paper-based TPT register was designed and programmed into Open Data Kit (ODK) , a mobile data collection application. Data quality was controlled by applying data validation checks in the ODK database system, training of research assistants who extracted data from TPT registers and continuous review of data captured into ODK.” (see page 10, lines 198-213).*

*How was missing data handled?*

*We added the following statement under data extraction section on how we handled missing or unlinked patients’ identifiers: “TPT data was linked to HIV care data using the patient HIV clinic identifier number (i.e. IDCNO). If the identifier was missing in the TPT paper-based register, then patient characteristics including; names, age, sex, home address, ART start date were used if available.” (see page 10, lines 202-204).*

*Also, under “Evaluation of TPT uptake and outcomes” section, we indicated that “…we additionally extracted prescribed drugs recorded in EMR to identify more individuals for whom TPT was prescribed during the study period in order to reduce on underreporting IPT uptake due to missing information” (see page 10, lines 186-189).*

*For handling missing data at analysis, we used a multiple imputation method and worst-case and best-case scenarios as sensitivity analysis approaches; the statement is under the statistical analysis section (see page 12, lines 243-245).*

*Key outcome definitions to be provided here (i.e. how was “TPT initiation” defined?)*

*Under statistical analysis section, we provided key outcome definitions for TPT uptake (i.e. TPT initiation) and TPT completion (see page 11, lines 223-226).*

9. Figure 1. Not clear if individuals are represented more than once in the diagram. I.e. if someone initiated IPT in 2016, would they additionally be eligible to initiate IPT in 2017 (and 2018, and 2019)? Again this comes back to the point about who is actually eligible to start TPT

***Response:*** *Thank you for the comment.*

*We have two scenarios highlighted below:*

*Scenario 1: If an individual had a clinic encounter anywhere between 2016 and in 2017, and was eligible for IPT but was not initiated on IPT in 2016, this individual would be included in the denominator for calculation of the proportion of IPT initiation in both 2016 and 2017*

*Scenario 2: If an individual had a clinic encounter between 2016 and in 2017, and was eligible for IPT and was initiated on IPT in 2016, this individual would not be counted in 2017.*

*Figure 1 has a footnote showing the number of patients who were excluded in the analysis for a specific year because they were on IPT prior. This includes both newly enrolled in the clinic and had prior history of IPT uptake and the continuing patients in the clinic who were initiated on IPT in the previous year.*

*To make this clearer, we have added a statement under the inclusion/exclusion section indicating “Individuals who were initiated on TPT in the previous calendar year were excluded in the analysis of subsequent years.” (see page 8, lines 153-157).*

10. Table 1: I am really unclear about “Total visited and eligible” and “Characteristics of eligible patients for TPT at beginning of each year.” What does “visited” mean? Are these unique people, or person-visits? What happened to people who were diagnosed with HIV/initiated ART (i.e. entered the cohort) during the year?

***Response:*** *We have corrected the confusing terms on Table 1.*

*The statements now read as “Total patients who had at least one clinic encounter during the year and were eligible for TPT”. This also accounts for patients enrolled into HIV care during the year. The counts presented are for unique patients.*

*We have also edited the footnote on Table 1 to indicate that “Characteristics of patients eligible for TPT at their first visit at the clinic in a specific year”.*

11. Table 1: I really struggle to believe that, in 2016, no people had previously been diagnosed with TB (especially given the data for the other years). Is this not a data collection issue?

***Response:*** *Thank you for the comment.*

*We agree that this is a data collection issue. The number of people previously diagnosed with TB for 2016 cannot be ascertained because data extraction was restricted to the period 2016-2019. We did not extract data prior 2016, thus TB history prior 2016 could not be ascertained.*

*We have dropped the TB history variable from the analysis because it could be accurately ascertained. We have updated tables 1, 2, 3 and the text accordingly.*

12. Table 1: In line with EQUATOR Guidelines, remove p-values. Given the large sample sizes here, these are probably not very meaningful.

***Response:*** *The p-values in Table 1 have been removed.*

13. How did uptake vary by clinic? What were the drivers and successes of differential uptake by clinic? Was this related to implementation of the campaign?

***Response:*** *Thank you for the comment.*

*TPT uptake did not significantly vary across clinics before and after the year of 100-days campaign. Implementation of the campaign was similar across the six clinics.*

*We have added a statement on TPT uptake across clinics in the results section (page 16, lines 289-290) and table provided in supplementary 2 (i.e.S2 Table 3a).*

14. Line 215-217: Again, what is the denominator here. As written, could be either unique participants, or clinic visits (with participants potentially having multiple visits). This really needs to be carefully and correctly described.

***Response:*** *These were unique participant in each year. A participant could only be carried over and counted in the denominator for the following year if he or she never initiated on TPT in previous year. The statement has been edited to avoid the confusion articulated by the reviewer (see page 16, lines 281-290).*

15. Although the impact of the campaign seems obvious, it would be more clearly reported if the authors could provide a figure (line-chart) showing the quarterly numbers of TPT initiations over the study period, with the campaign period indicated. As the campaign only during the last study year, this figure would then have sufficient resolution to show the impact of the campaign.

***Response:*** *We agree. A line graph for TPT uptake indicating the campaign period has been plotted and included in supplementary 2, and labelled “S2 Fig 1”. Also, a statement referencing this has been added in the results section (see page 16, line 290).*

16. Lines 238-239: “Stratified analysis indicated TPT uptake was similar across age groups and sex of participants over the 4 years studied (Fig 2a and 2b)”. This is not what is shown by the regression analysis in Table 2, with adolescents aged 15-19 years having substantially higher IPT uptake compared to older aged people.

***Response:*** *Thank you for the comment.*

*Stratified analysis was performed by fitting a regression model with interaction between calendar years and selected participants’ characteristics to compare trends of TPT uptake across covariates’ groups overtime. E.g. stratified analysis was to answer a question like, “Was the trend of TPT uptake different across sex over the 4 years?”. Whereas, results presented in Table 2 are for the main effects (i.e. overall effect) of the characteristics on TPT uptake. i.e. estimates from Table 2 can answer a question like “On overall, is there a difference in TPT uptake across sex of the participant?”. So, stratified analysis addresses a question on trend whereas main effects (i.e. Table 2) address overall effect of the covariate.*

*We have edited the statement on stratified analysis results to indicate that results apply trends over the 4 years studied (see page 17, lines 304).*

17. Table 3: There are substantial missing data here, both for outcomes and denominators. In the methods, the authors state that they used multiple imputation by chained equations, but this doesn’t seem to be the case? Given the risk of misclassification bias, the authors should report complete case analysis, as well as imputed analysis and best/worst case scenario analysis for handling missing data.

***Response:*** *Thank you for the comment.*

*Sensitivity analyses for handling missing data i.e. multiple imputation, worst-case and best-case scenarios have been performed and a statement has been added in results section (see page 18, lines 331-335). Also, detailed results on all covariates in models are presented in supplementary 2, table 4.*

18. Lines 204-306: “This was achieved using existing health care resources, system strengthening, multi-stakeholder engagement in the campaign, and enhanced TPT delivery, monitoring and reporting”. This is unfortunately insufficiently clear. Readers planning similar interventions would not be able to replicate the success of the Uganda programme without considerably more detail about what the campaign actually involved.

***Response:*** *Thank you for the comment.*

*An additional paragraph explaining what was involved in the campaign has been added in methods section (see page 8, lines 134-146, and page 9, lines 171-184). For more detailed information about the implementation of the 100-days campaign, we provided reference #18 (i.e. document on “100-Day Accelerated Isoniazid Preventive Therapy Scale Up Plan, 2019”). Additionally, in supplementary 2, we provided the outline of key activities and their timelines during the campaign.*

19. Discussion needs much greater description (likely 1-2 paragraphs) of the potential benefits and harms to individuals and programmes should this “campaign” approach be adopted routinely in Uganda and elsewhere.

***Response:*** *Thank you for the comment.*

*An additional paragraph on potential benefits and harms to individuals, has been added in the under the discussion section (see page 20, lines 383-390).*

20. In the response to the Editor, the authors state that dataset and code for replication are available as Supplemental Material, in line with PLOS One requirements - they are not.

***Response:*** *Thank you for the comment.*

*The dataset has now been provided as supplementary material, and labelled as “supplementary 1 _tpt_data_PLosOne”.*

Minor comments

1. Line 33: reword “reactivation, probably to “disease incidence” or similar

***Response:*** *Thank you for the comment.*

*The statement has been reworded to “Tuberculosis preventive therapy (TPT) effectively decreases rates of developing active tuberculosis disease in people living with HIV (PLHIV) who are at increased risk.” (see page 3, lines 32-34).*

2. Figure 1: exclusions might be better show using branching boxes, to add clarity to the flow of the diagram.

***Response:*** *Thank you for the comment.*

*Figure 1 has been edited with exclusions indicated as branching boxes.*

3. Line 214 (and throughout next paragraph): “Prevalence” is probably not correct here. Suggest remove to read “Trends in TPT uptake”

***Response:*** *Thank you for the comment.*

*The paragraph has been edited and term “prevalence” has been removed.*

**Reviewer #2:** This paper reports on the impact of Uganda's 100-day campaign to scale-up TPT in PLHIV. The results are impressive. The authors have responded to the previous reviewers' comments. I have a few additional comments/questions for the authors to consider:

1- Study population: It would be helpful to clarify the inclusion/exclusion criteria a bit more. Would a patient have been included if they visited a clinic multiple times within a year if they were eligible for TPT at one visit but ineligible at a subsequent visit? For example, if a PLHIV presented for care and was eligible for TPT, and later that year returned to clinic and was ineligible (e.g., diagnosed with TB), would they have been included in the analysis?

***Response:*** *Thank you for the comment.*

*If a patient was eligible at start of the current year but during the course of the year became ineligible e.g. diagnosed with TB, this patient could be considered in the denominator for the current year but excluded in denominators in the subsequent years.*

*Additional statement had been added in the inclusion and exclusion criteria as guided (see page 8, lines 153-157).*

2- There appears to be a typo in the legend for Figure 1. The number reported to have been diagnosed with TB/on TB treatment is the same for 2016-2019 (n=1,168)

***Response:*** *Thank you for the comment.*

*The numbers “diagnosed with TB/on TB treatment” have been corrected.*

3- The numbers "visited and eligible" in Table 1 do not match the numbers eligible in Figure 1.

***Response:*** *Thank you for the comment.*

*Table 1 Included only participants who appeared in the EMR since it was the source document with detailed participants’ characteristics.*

*Number of eligible participants presented in table 1 compared to those in figure 1, are less by number of participants who were in paper-based TB registers but their IDs could not be linked with HIV data in the EMR, i.e. 22, 30, 63, 380 in 2016, 2017, 2018 and 2019 respectively.*

*A footnote has been added below table 1 with an explanation of this difference.*

4- Do the authors have an explanation for the decline in TPT uptake in 2018? This would be interesting to include in the discussion.

***Response:*** *Thank you for the comment.*

*The decline in TPT uptake in 2018 was due to general stockouts at the National Medical Stores and health facilities were affected.*

5- The authors state "as expected in 2019 uptake increased dramatically by about 25% (relative increase) from 2.5% in 2018 to 64.8% in 2019." I find this confusing -- the increase from 2.5% to 64.8% is many-fold. Could the authors clarify?

***Response:*** *The increase was ~26 fold comparing TPT uptake in 2018 compared to 2019 (64.8/2.5 = 25.92) and has been reported as such. Thanks for pointing out this error.*

6- Statement that "TPT uptake was similar across age groups and sex of participants over the 4 years studied" seems contradicted by the data presented in Table 2.

***Response:*** *Thank you for the comment.*

*Stratified analysis was performed by fitting a regression model with interaction between calendar years and selected participants’ characteristics to compare trends of TPT uptake across covariates’ groups overtime. E.g. stratified analysis was to answer a question like, “Was the trend of TPT uptake different across sex over the 4 years?”. Whereas, results presented in Table 2 are for the main effects (i.e. overall effect) of the characteristics on TPT uptake. i.e. estimates from Table 2 can answer a question like “On overall, is there a difference in TPT uptake across sex of the participant?”. So, stratified analysis addresses a question on trend whereas main effects (i.e. Table 2) address overall effect of the covariate.*

*We have edited the statement on stratified analysis results to indicate that results apply trends over the 4 years studied (see page 17, lines 304).*

7- The discussion states that the evaluation was done 3 months after implementation of the 100-day TPT campaign. Doesn't this mean it was too early to evaluate TPT completion (which takes 6-months) for much of the 2019 study population?

***Response:*** *Thank you for the comment.*

*The main aim of the 100-day IPT accelerated campaign was to initiate 300,000 PLHIV on IPT from July 2019 to September 2019 and achieve IPT completion rates of 100% of individuals previously initiated on TPT in quarters: October – December 2018, and January – February 2019. So, TPT completion was evaluated only among those who previously initiated on TPT for ≥6 months prior to the campaign.*

*We have edited statement in the discussion section first paragraph (see page 20, lines 375-379) to clearly show the main aim of the campaign in terms of TPT completion was to achieve 100% TPT completion among those initiated ≥6 months prior the campaign. Additionally, we have edited the definition for TPT completion under statistical analysis section (see page 11, lines 224-226).*

**Reviewer #3:** Thank you for the opportunity to review this manuscript. The authors present findings from an evaluation of a TPT uptake campaign in Uganda. Major revisions are necessary before I would recommend this manuscript for publication.

Major comments:

1- Line 98 indicates that this is a ‘cross-sectional review’ – however the statistical analysis is for a longitudinal study design and line 160 notes that there were ‘repeated observation of participants over the 4 years.’ I think cross-sectional isn’t the correct terminology for this paper. Would suggest ‘retrospective cohort study.’

***Response:*** *Thank you for the comment.*

*We have re-written the statement and indicated that it was cohort study design that involved retrospective review of patients’ medical records (see page 6, line 101).*

2- I would strongly recommend a more granular presentation of this data into quarters or months – given that the 100 day campaign was Q3 of 2019 (July-Sept), it would be informative to see if TPT uptake immediately declined in Q4 or if high levels of TPT prescribing were maintained

***Response:*** *Thank you for the comment.*

*A line graph for TPT uptake in quarters has been plotted and included in supplementary 2, and labeled “S2 Fig 1”. Also, a statement referencing this has been added in the results section (see page 16, line 290).*

3- It’s unclear why January 2016 was chosen as the starting point of the study implementation period. Can the authors provide further insight into why the specific time frame was chosen?

***Response:*** *Thank you for the comment.*

*We arbitrarily chose January 2016 as the starting point for our evaluation, because a health workers’ guide for provision of isoniazid preventive therapy to PLHIV was rolled out in June 2014 and we wanted to allow enough time for the information to be disseminated and implemented. Therefore, we reasoned that one and half years after rolling out the IPT guide, health workers would have mastered the guide and the IPT program implementation would running effectively.*

*An additional statement to reflect this has been added under the inclusion in the methods section (see page 9, lines 158-163).*

4- Line 113 states that the study included all ‘asymptomatic’ PLHIV aged 15 years and above. How was symptom status determined? Were the presence or absence of symptoms always recorded (i.e. if a patient had no symptoms, was it clearly stated in the medical record? Or was the absence of documentation of symptoms interpreted as an indication of no symptoms?)? If a patient had multiple visits over a 1 year period and were asymptomatic at the first visit but symptomatic at visit 2, were they included? This could be a major source of misclassification if symptom status was not well documented.

***Response:*** *Thank you for the comment.*

*If a patient was eligible at the start of the current year but during the course of the year (ie. On a subsequent visit within the year) became ineligible e.g. became symptomatic or diagnosed with TB, this patient could be considered in the denominator for the current year but excluded in denominators in the subsequent years. An additional statement had been added in the inclusion and exclusion criteria as guided (see page 8, lines 154-157).*

*Given the retrospective nature of the study, we could only use the documented information to classify the presence or absence of symptoms. We agree that there is high likelihood of misclassification especially if symptom status was not documented. This has been acknowledged under limitation.*

5- Lines 189-190 indicate that data were missing for 10% and 4% of patients for TPT uptake and completion, respectively. However, in Figure 1, 26,478 patients initiated on IPT in 2019 but completion was assessed for only 7,713 which would mean that completion data is missing for more than 70% of those who initiated IPT in that year. This raises concerns regarding the validity of the completion analyses and results presented. The authors should provide further detail into why 70% of patients who initiated IPT in 2019 could not be found in the EMR.

***Response:*** *Thank you for the comment.*

*The high proportion of unlinked TPT register and HIV care data was because some patients’ HIV clinic identifiers that were used link HIV care and TPT data were either missing or wrongly captured in the paper-based TPT registers. Also, since TPT is only offered for a short time i.e. 6 months for isoniazid monotherapy, and that most patients received a full six-months course at once, it could be hard to reconcile the wrongly entered identifiers in the paper-based IPT registers with those in the ART clinics. However, when we compared the characteristics of patients whose TPT data and HIV care data was linked versus those whose data was not linked, they were similar. This implies that there was little potential effect of selection bias.*

*In the manuscript, we explained this in discussions section under limitations (see page 22, lines 429-435).*

6- Line 314 of the discussion states that the national guidance prior to 2018 was to initiate only the newly enrolled PLHIV on TPT. This is an important point as that indicates that only new ART initiates were eligible for TPT prior to that change. Figure 1 should be stratified by new vs stable ART clients in order to present TPT uptake based on ART status.

***Response:*** *Thank you for the comment.*

*There is information on TPT uptake stratified by ART status presented in supplementary material 2, table* *S2 Table 2 Proportion of TPT uptake across participants characteristics by year.*

Minor comments:

7- The abstract needs section headers (introduction, methods, results, conclusion)

***Response:*** *Thank you for the comment.*

*According to the PLOS ONE guidelines, there are no section headers in the abstract.*

8- Lines 77-79 reference two different WHO global TB reports (2018 and 2020). For consistency, please use numbers/figures from the 2020 report. You can consider re-phrasing this section as, “Uganda is among the WHO’s 30 high TB/HIV burden countries which contribute about 60% of the total TB/HIV burden globally. In 2019, approximately XX% of notified TB patients were living with HIV and 6.7% of PLHIV newly enrolled in care were diagnosed with TB. [reference]”

***Response:*** *Thank you for the comment.*

*The statement has been rephrased and the statistics and reference has been added (see page 5, lines 74-76).*

9- Lines 79-81: when did the Uganda MoH start recommending TPT for PLHIV? Please add the year to this statement. For example, you could re-phrase as: “Since 20XX, the Uganda Ministry of Health (MoH) has recommended that all PLHIV without symptoms suggestive of active TB disease receive TPT regardless of CD4 count, ART status, history of TB treatment, and pregnancy status.”

***Response:*** *Thank you for the comment.*

*The statement has been revised accordingly, and the year when the Uganda MoH started recommending TPT for PLHIV has been indicated (see page 5, lines 77-79).*

10- Lines 100-101 describe the 6 health facilities from which patient medical records were abstracted and notes that 5 were health center level III and 1 was a health center IV. Can you add additional detail about what it means for a facility to be level 3 vs level 4? Are there different services provided? Different operating hours? Different staffing levels and/or cadres of staff (i.e. nurses only vs nurses and doctors?)?

***Response:*** *Thank you for the comment.*

*We have provided some details on services provided at health facility levels 3 and 4 (see page 7, lines 109-112).*

11- Line 142 states that the data collection tools are presented in the appendix but I couldn’t find them. Please upload them or if they’re not available, remove this sentence.

***Response:*** *Thank you for the comment.*

*The data collection tools are now provided as supplementary 3.*

12- The box at the bottom of Figure 1 indicates reasons for patients not being eligible for IPT and lists the exact same number of people (1,168) for each year as diagnosed with active TB or on TB treatment. Please review those numbers for accuracy.

***Response:*** *Thank you for the comment. These were typos, now corrected.*

13- Line 218 indicates that there was a 25% relative increase in TPT uptake from 2018 to 2019 but it is a 25 times increase, not 25%.

***Response:*** *The increase was ~26 fold comparing TPT uptake in 2018 compared to 2019 (64.8/2.5 = 25.92) and has been reported as such. Thanks for pointing out this error.*

**Reviewer #4**: This study reports the change in the TPT uptake in 6 Uganda's clinics after a nation-wide campaign for TPT upscale in Uganda. The results are very impressive, and I enjoyed really reading the manuscript.

Comments:

1. I appreciate authors using the GEE model that accounts for correlated observations both within clinic and between the same patients across years.

***Response:*** *Thank you for the comment.*

2. Introduction (line 82): authors report TPT uptake of 16-17% before 2019, but their study results (line 217) show only 2.5-4.5%. I wonder whether the patients in the sampled clinics are somehow different from general population, or this is not the same metric that being reported.

***Response:*** *Thank you for the comment.*

*There are number of differences in population definitions between the quoted study (the statistics we present in the introduction) and our study.*

*1) Different clinics were studied.*

*2) The quoted study included only PLHIV newly registered for ART in 2017 whereas in our study we included both newly enrolled and ART experienced PLHIV. In our study we observed a slightly higher TPT uptake before the 100-day campaign for PLHIV newly enrolled in HIV care/newly started on ART compared to the ART experienced, which seems to agree with the TPT uptake documented in the quoted study. As explained in the discussion, in programmatic setting, clinics tended to prioritise TPT initiation to PLHIV newly enrolled in HIV care than the ART experienced when there was limited TPT stock.*

*3) Also, the quoted study only sampled on 400 PLHIV from the selected clinics whereas in our study we analysed had everyone that was eligible for TPT.*

3. Introduction (line 86): Authors state the campaign was only one of interventions. What were the others, and could they be also responsible for the increase in the TPT uptake?

***Response:*** *Thank you for the comment.*

*Yes, the 100-day accelerated Isoniazid preventive therapy scale-up campaign was the only campaign conducted during the period. We therefore believe that the increase in the TPT uptake we observed was due to this campaign.*

4. Methods (line 98): this is not a cross-sectional study. I think it would be best described as a (living) cohort study.

***Response:*** *Thank you for the comment. We have re-written the statement and indicated that it was cohort study design that involved retrospective review of patients’ medical records (see page 7, line 101).*

5. Methods (line 100): It should be clarified whether (if) these 6 facilities were all part of the campaign.

***Response:*** *Thank you for the comment.*

*A sentence has been added under setting in methods section (see page 8, line 143).*

6. Methods, outcome: TPT uptake - prevalence vs. incidence. I found the usage of the phrase "prevalence of TPT uptake" a bit misleading, as prevalence typically refers to proportion of all patients with event and "uptake" typically refers to new cases (eg as in incidence). I would recommend authors to make it more clear that the outcome was newly initiated TPT in each year (if that was indeed the case).

***Response:*** *Thank you for the comment.*

*We have edited the definition of TPT uptake to read as newly initiated on TPT among PLHIV in HIV care who were eligible for TPT in a specific year (see page 11, lines 223-224).*

7. Methods, model: As there is a natural yearly fluctuation in TPT uptake, authors could consider comparing the TPT uptake in 2019 versus all previous years (2016-2018) combined, rather than comparing to individual years; 2016 (line 220), or 2018 (line 221).

***Response:*** *Thank you for the comment.*

*We agree that the analysis of combined previous years (i.e. 2016-2018) compared to 2019, would be interesting, however, the aim of the study was to compare trends overtime and the impact of the 100-day campaign on these trends. Also, combining all the previous years (2016-2018) may not account the variations in implementation of the TPT program across the 3 years.*

8. Results (line 218): reported change in uptake from 2.5% to 64.8% does not correspond to 25% increase, but rather a 26-fold increase. It is then later stated correctly (eg line 221).

***Response:*** *The increase was ~26 fold comparing TPT uptake in 2018 compared to 2019 (64.8/2.5 = 25.92) and has been reported as such. Thanks for pointing out this error.*

9. Typo (line 54): should be 82.6%

***Response:*** *Thank you for the comment. The typo has been corrected.*

**Thank you**

**Joseph Musaazi**

**Corresponding Author**
