# Supplementary material for: Increased uptake of tuberculosis preventive therapy (TPT) among people living with HIV following the 100-days accelerated campaign: A retrospective review of routinely collected data at six urban public health facilities in Uganda
Source: PLoS One. 2023 Feb 23;18(2):e0268935. doi: 10.1371/journal.pone.0268935 (PMC9949662; doi:10.1371/journal.pone.0268935)
Supplement: Attachment — Submitted filename: Response to Reviewers.docx [file pone.0268935.s007.docx]

The Editor,

PLoS One

Date: 19^th^ January 2023

Dear Sir,

**RE: RESPONSE TO REVIEWERS’ COMMENTS MANUSCRIPT ID: PONE-D-22-13680R2 “Increased uptake of tuberculosis preventive therapy (TPT) among people living with HIV following the 100-days accelerated campaign: A retrospective review of routinely collected data at six urban public health facilities in Uganda”.**

*The authors of the reviewed manuscript thank the academic editor and reviewers for their consideration and careful review of the manuscript. Below, we provide detailed point-by-point responses to the reviewers’ comments in maroon font and italics. We have also tracked the changes in the revised manuscript.*

*In this document, we have also provided page and line numbers as referenced from the tracked manuscript to enable reviewers easily track the changes made as per their comments.*

**Additional Editor Comments:**

Many thanks for the first revisions. As seen below, Reviewer 3 has still some critical concerns around the analysis.

[Note: HTML markup is below. Please do not edit.]

**6. Review Comments to the Author**

Please use the space provided to explain your answers to the questions above. You may also include additional comments for the author, including concerns about dual publication, research ethics, or publication ethics. (Please upload your review as an attachment if it exceeds 20,000 characters)

**Reviewer #1**: Thanks to the authors for a comprehensive response, which has strengthened the manuscript. I have no other questions.

***Response****: Thank you.*

**Reviewer #2**: The authors have responded to all of the queries. I have two final thoughts:

- Line 158: The authors can remove "arbitrarily," which has a negative connotation. There is rationale for the starting point of the evaluation.

***Response****: Thank you for the comment.*

*The word “arbitrarily” has been removed. See page 9, line 168.*

- Analysis/Discussion: The TPT completion rates are extremely high. Though promising, I worry this could be partially due to bias from only using the EMR records (which comprised only approximately 30% of the TPT initiation from 2019). The authors address this is the discussion, but conclude that there was unlikely to be bias. I am less certain, and think the results warrant a bit more caution in their interpretation.

***Response****: Thank you for the comment.*

*The statement referred to in the discussion under limitations has been removed. See page 28, line 557-559.*

**Reviewer #3:** Thank you for the opportunity to review this revised manuscript. The authors present findings from an evaluation of a TPT uptake campaign in Uganda. Further revisions are necessary before I would recommend this manuscript for publication.

Major comments:

- Given that the short duration of the campaign and the focus on completion for patients initiated in Q4 2018 and Q1 2019, I would suggest that the primary analysis be conducted quarterly rather than annually (as opposed to the quarterly data being presented only in the appendix). Or is there a statistical reason to focus on annual data for the primary analysis?

***Response****: Thank you for the comment.*

*We agree with your recommendation.*

*The analysis has been repeated and presented quarterly data as the primary analysis.*

- On lines 392-394 of the discussion, the authors indicate that national guidance before 2018 was to enroll only the newly enrolled PLHIV in care and TPT eligibility was expanded in 2019. In figure 1, it’s indicated that 38,704 patients were eligible for TPT in 2016, for example. Is this correct? Are these only newly enrolled? If this is all PLHIV, I would suggest that the primary analysis be focused only on newly enrolled PLHIV since they were the only patients eligible until the last year covered by this analysis. Data on established ART clients could be presented in a supplementary appendix.
- Figure 1 and table 1 have slightly different numbers – as per the footnote, table 1 excludes individuals who were in paper based registers but not the EMR. Can figure 1 be revised so that the data across figure 1 and table 1 are consistent?

***Response****: Thank you for the comment.*

*According to the national TPT guidelines, all PLHIV in care were eligible for TPT (See ref#14). However, in instances when there was TPT shortages at the health facilities, the national guidance to the health facilities was to prioritize TPT initiation to the PLHIV not yet on ART due to their vulnerability in terms of acquiring active TB compared the ART established clients. Nevertheless, all PLHIV could be initiated on TPT if sufficient stock was available. Between 2018 and 2019, there was an increased TPT supplies at the national level, and such shortages were minimized. This provides the reason for higher TPT uptake among those “not on ART” compared to the “ART established clients” before 2018, which was leveled after the 100-day accelerated TPT campaign. We have edited the statement in the discussion to read better and have consistency. See page#26, lines 505-510.*

*Figure 1 has been revised so that the data are consistent with that in table 1. On fig 1, we have inserted another layer (i.e. Eligible & analyzed for TPT uptake) to show the numbers patients actually analyzed for TPT uptake per year after excluding those who appeared in TPT paper-based register but had no information the EMR. See pages #14-15.*

Minor comments:
- On lines 89-91 (and again on lines 136-137), the authors describe the 100 day campaign and indicate that completion was focused on patients who initiated in the quarters October-December 2018 and January-February 2019. Should the second quarter be January-March 2019 (to reflect a 3 month period?)?

***Response****: Thank you for the comment.*

*We agree, the second quarter targeted for 100% TPT completion in the 100-day should be January-March 2019 to reflect the 3 months period. This was however stated as January-February 2019 in the referenced document – 100-Day Accelerated Isoniazid Preventive Therapy Scale Up Plan (ref#18).*

*To make the quarter complete, we have edited the statements to indicate it as January-March 2019. See page #6, line# 101, page#8, line# 147, and page# 25, lines 485 – 489.*

- On line 110, the comma after ‘inpatient health’ should come after the word ‘services’

***Response****: Thank you for the comment.*

*The comma has been moved. See page# 7, line 120.*

- Line 256 states that 95% of patients were eligible for TPT during each year. However, in 2018, it was 91% (40,390/44,439) and in 2019, it was 89% (40,867/45,868). Line 256 should be revised to reflect the data presented in figure 1.

***Response****: Thank you for the comment.*

*The statement has been revised with correct value to match data presented in fig1. See page #14, lines 283.*

- On lines 258-259, the authors state that ‘data were missing on 10% and 4% of patients for TPT uptake and completion analyses, respectively.’ However, 26,478 people initiated TPT in 2019 and only 7,713 were evaluated for completion. So isn’t completion data missing for 71% of individuals who initiated TPT in that year?

***Response****: Thank you.*

*A statement has been added to show the 70% data missingness on TPT outcomes for individuals excluded from TPT completion analysis due to the unmatched IDs from the electronic HIV care database and paper-based TPT registers. See page #14, lines #284 – 289.*

- In the response to reviewer #2 (point 4), the authors indicated that the decline observed in 2018 was due to stockouts at the national medical stores but I didn’t see this reported in the revised manuscript. This could be added to the discussion to provide more context for the results.

***Response****: Thank you for the comment.*

*A statement on the cause of the TPT uptake decline in 2018 has been added in the discussion section. See page #25, lines #489 – 493.*

- While the discussion states that national guidance was to provide TPT to only those newly enrolled in care through 2018, the introduction (lines 77-79) and the methods (lines 120-122) state that the MoH recommended all PLHIV receive TPT regardless of ART status. Likewise, lines 143-144 states ‘the same national eligibility criteria for TPT initiation before the campaign, were maintained.’ But based on what’s written in the discussion, eligibility was expanded from only those newly enrolled in care to all ART clients, correct? Would suggest reviewing these statements for consistency.

***Response****: Thank you for the comment.*

*The statement in discussion has been revised to have consistency.*

*In the program setting, the guidance to health facilities providing TPT was to prioritize TPT for newly enrolled in care “not on ART” in situations of drug stock-outs. However, all PLHIV were eligible and could be initiated on TPT when drugs were available. See page#26, lines 506-513.*

- Lines 177-179 state that PLHIV initiated on TPT were provided with monthly refills and follow-up until they had completed 6 months of treatment. However, lines 127-129 state that TPT refill visits are aligned with ART refill visits and are given for either 1 month, 3 months, or 6 months. Would suggest reviewing these statements for consistency.

***Response****: Thank you for the comment.*

*The monthly TPT refills and follow-up until completion of a 6-months treatment course was the guideline, however, in a programmatic setting, the practice is to provide TPT refills on the same day of ART refill visit. This helps to achieve TPT adherence and also reduces the number of times an individual has to visit the clinic which can be costly and time consuming.*

*A statement with additional explanation has been added in the treatment procedure section. See page #9, lines #187 – 188, and page #10, lines #189 – 192.*

Reviewer #4: (No Response)

***Response****: Thank you.*

Reviewer #5: General comments: I have some questions and suggestions for this manuscript that will hopefully improve the communication of how analyses were implemented and improve the reader's understanding.

As a non-statistical comment, it would be really interesting to see one more year of data to see if these gains were sustained.

***Response****: Thank you for the comment.*

*We agree, it would be interesting if more years of data are added to examine if these gains were sustained. However, the objective of this manuscript was to evaluate whether the 100-days of accelerated campaign achieved its objectives: (1) increasing TPT uptake from about 30% to about 50% during the 100-days of accelerated TPT initiation i.e. from July 2019 to October 2019. (2) achieve 100% completion until September 2019 among PLHIV initiated on TPT during the quarters of October - December 2018 and January – March 2019.*

*We have added a statement recommending future evaluation to assess sustainability of these gains with more years of data post the 100-days accelerated TPT intervention. See page #29, lines #576 – 578.*

Specific comments:
1. (lines 223-242) The experimental unit of these analyses is unclear to me. Did you analyze data at the individual level or aggregated numbers from facilities? Said differently, is your outcome in these models a binary yes/no of TPT uptake or TPT completion or is the number of TPT uptakes or TPT completions at the facility (hopefully with an offset included in the regression model)? Based on lines 235-237, I am pretty sure this is an individual-level model, but it would be good to make this explicit.

***Response****: Thank you for the comment.*

*Data was analyzed on individual level not aggregated numbers. TPT uptake or TPT completion were entered as binary (yes/no) outcomes when fitting the Poisson models with robust error variance.*

*A statement has been added in statistical methods section to make it clear the unit of analysis. See page #12, lines #241 – 242.*

2. (Ignore if individual-level model) If you have a count-based model, did you check for overdispersion? Overdispersion can be a big problem in Poisson regression and I strongly urge the authors to move a negative binomial model or demonstrate that there is no overdispersion.

***Response****: Thank you for the comment.*

*Data was analyzed on individual level not aggregated as indicated in response to the previous comment.*

3. I am curious about the choice of Poisson over log binomial for binary outcomes. It is possible and, if done correctly (which I think you have), acceptable, e.g., <https://doi.org/10.1093/aje/kwh090>, but I am curious about the choice. I think my confusion in the previous couple comments centers around that choice.

***Response****: Thank you for the comment.*

*The choice of Poisson regression with robust variance estimates, over log-binomial is because the former directly and appropriately estimates relative risk whereas the latter often face a convergence problem which requires proper choice of starting values.*

*A statement on justification for the choice of the model used has been added. See page #12, lines #257 – 259.*

4. (lines 227-230) Please provide a methodological citation for GEE.

***Response****: Thank you for the comment.*

*A citation for GEE has been provided (reference#26). See page #12, lines #248 – 251.*

5. (lines 230-231) This statement is incorrect. The odds ratio does not overstate the association. If it did, there was no way it would be well accepted. What you mean to say is that the odds ratio overestimates the *risk* in outcomes that are prevalent, which is a known fact. That is due to flaws in how people interpret the results, not the method itself.

***Response****: Thank you for the comment.*

*The statement has been edited. See page #12, lines #252 – 253.*

6. (lines 232-233) This seems redundant with the previous sentence. Maybe I am missing something?

***Response****: Thank you for the comment.*

*The statement referred to in this comment explains the model used for examining factors associated with TPT completion, while the previous statement in the same section explains the model for examining factors associated with TPT uptake. See page #12, lines #248 – 257.*

7. (lines 243-245) Please provide methodological citations for all methods used here, e.g., VIFs, MICE, etc.

***Response****: Thank you for the comment.*

*Citations have been provided. VIFs (reference#29), MICE (reference#30), and best-worst case scenario (reference #31). See page #13, lines #270 – 272.*

8. (Figures 2-5) Please describe in the methods section how the predicted probabilities are computed.

***Response****: Thank you for the comment.*

*A statement has been added on how the predicted probabilities were computed. See page #13, lines #267 – 269.*

9. (Figures 2-5) It would also be good to shift the points slightly in order to better see the confidence interval overlap. For instance, in Figure 4a, it's impossible to tell which CIs are overlapping. Unfortunately, I don't know how to do this in Stata. In R's ggplot package, this can be done with the position_dodge function.

***Response****: Thank you for the comment.*

Plots have been redone in “R” using position_dodge function to shift points a bit and be able to see confidence interval overlap. *See pages # 31-38*.
