# Supplementary material for: Increased uptake of tuberculosis preventive therapy (TPT) among people living with HIV following the 100-days accelerated campaign: A retrospective review of routinely collected data at six urban public health facilities in Uganda
Source: PLoS One. 2023 Feb 23;18(2):e0268935. doi: 10.1371/journal.pone.0268935 (PMC9949662; doi:10.1371/journal.pone.0268935)
Supplement: Attachment — Submitted filename: Response to Reviewers.docx [file pone.0268935.s008.docx]

The Editor,

PLoS One

Date: 01^st^ February 2023

Dear Sir,

**RE: RESPONSE TO REVIEWERS’ COMMENTS MANUSCRIPT ID: PONE-D-22-13680R3 “Increased uptake of tuberculosis preventive therapy (TPT) among people living with HIV following the 100-days accelerated campaign: A retrospective review of routinely collected data at six urban public health facilities in Uganda”.**

*The authors of the reviewed manuscript thank the academic editor and reviewers for their consideration and careful review of the manuscript. Below, we provide detailed point-by-point responses to the reviewers’ comments in maroon font and italics. We have also tracked the changes in the revised manuscript.*

*In this document, we have also provided page and line numbers as referenced from the tracked manuscript to enable reviewers easily track the changes made as per their comments.*

**Additional Editor Comments:**

Thank you for submitting your manuscript to PLOS ONE. After careful consideration, we feel that it has merit but does not fully meet PLOS ONE’s publication criteria as it currently stands. Therefore, we invite you to submit a revised version of the manuscript that addresses the points raised during the review process.

There is one small comment from Reviewer 3 left (description of the 100 day campaign). Please address as soon as possible. Once addressed, an editorial decision will be taken (the manuscript will not be re-reviewed).

**6. Review Comments to the Author**

**Reviewer #3:** Thanks for the revisions to your paper. I have one last comment (please note, it is not necessary for me to re-review this paper in light of this comment):

In my previous review, I had noted that the 100 day campaign was described as focusing on patients who initiated in quarters Oct-Dec 2018 and Jan-Feb 2019 and asked whether the second quarter should reflect Jan-Mar 2019. In the authors reply, they indicated that they had changed the text to reflect a full quarter but that the 100 day campaign reference document was focused on Jan-Feb 2019. I would recommend describing the campaign as it was implemented. If the focus was truly on Jan-Feb 2019 patients, I would state that so that your manuscript is accurately reflecting the Ministry of Health’s program.

***Response****: Thank you for the comment.*

*We have made edits and indicated the period the 100-day campaign targeted for TPT completion (i.e. initiated in quarters Oct-Dec 2018 and Jan-Feb 2019) as stated in the 100-day campaign reference document.*

*See page #6 line 95, page #8 line 141, and page #24 line 465.*
